# Lab-on-the-Needles: A Microneedle Patch-Based Mobile Unit for Highly Sensitive *Ex Vivo* and *In Vivo* Detection of Protein Biomarkers

**DOI:** 10.1021/acsnano.4c11238

**Published:** 2025-01-07

**Authors:** Ying-Pei Hsu, Nan-Si Li, Hao-Han Pang, Yu-Chi Pan, Hung-Pei Tsai, Hsiao-Chien Chen, Ying-Tzu Chen, Chen-Hsun Weng, Shiao-Wei Kuo, Hung-Wei Yang

**Affiliations:** 1 Department of Materials and Optoelectronic Science, National Sun Yat-sen University, Kaohsiung 80424, Taiwan; 2 Department of Biomedical Engineering, National Cheng Kung University, Tainan 70101, Taiwan; 3 Graduate Institute of Medicine, College of Medicine, Kaohsiung Medical University, Kaohsiung 80708, Taiwan; 4 Division of Neurosurgery, Department of Surgery, Kaohsiung Medical University Hospital, Kaohsiung Medical University, Kaohsiung 80708, Taiwan; 5 Center for Reliability Science and Technologies, Chang Gung University, Taoyuan 33302, Taiwan; 6 Kidney Research Center, Department of Nephrology, Chang Gung Memorial Hospital, Linkou, Taoyuan 33305, Taiwan; 7 Department of Neurosurgery, Chang Gung Memorial Hospital, Linkou, Taoyuan 33305, Taiwan; 8 Medical Device Innovation Center, National Cheng Kung University, No. 1, University Rd., Tainan City 70101, Taiwan; 9 Department of Medicinal and Applied Chemistry, Kaohsiung Medical University, Kaohsiung 80708, Taiwan

**Keywords:** microneedle patch (MNP), zeolitic imidazolate framework-8 (ZIF-8), liquid biopsy, transdermal detection, mobile healthcare testing (MHCT)

## Abstract

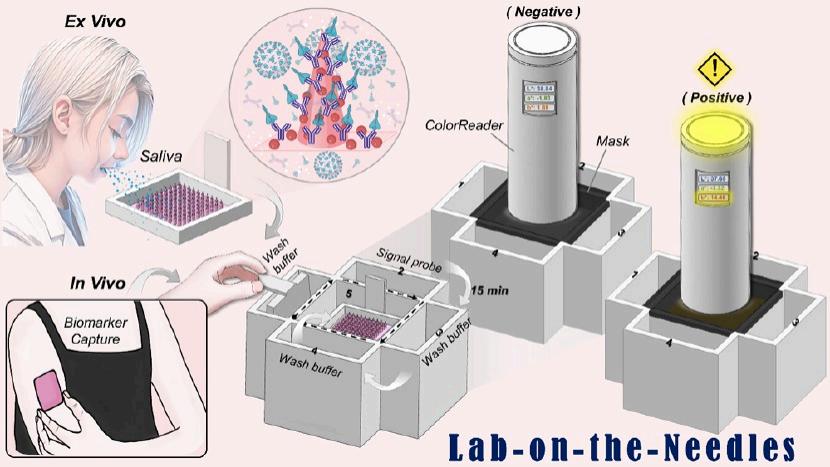

Detection of biomarkers associated with physiological conditions provides critical insights into healthcare and disease management. However, challenges in sampling and analysis complicate the detection and quantification of protein biomarkers within the epidermal layer of the skin and in viscous liquid biopsy samples. Here, we present the “Lab-on-the-Needles” concept, utilizing a microneedle patch-based sensing box (MNP-based SenBox) for mobile healthcare applications. This system facilitates the rapid capture of protein biomarkers directly from the *in situ* epidermal layer of skin or liquid biopsies, followed by on-needle analysis for immediate assessment. The integration of horseradish peroxidase-incorporated zeolitic imidazolate framework-8 (HRP@ZIF-8) as a sensitive and stable signal probe, the detection limit for anti-SARS-CoV-2 NP IgA antibodies and various SARS-CoV-2 S1P mutant strains improves by at least 1,000-fold compared to FDA-approved commercial saliva lateral flow immune rapid tests. Additionally, the MNP-based SenBox demonstrated minimally invasive monitoring and rapid quantification of inflammatory cytokine levels (TNF-α and IL-1β) in rats within 30 min using a portable ColorReader. This study highlights the potential of the MNP-based SenBox for the minimally invasive collection and analysis of protein biomarkers directly from *in situ* epidermal layers of skin or liquid biopsies that might facilitate mobile healthcare diagnostics and longitudinal monitoring.

## Introduction

The viscosity of liquid biopsy solutions can significantly influence the detection efficiency of biosensors. In solutions with higher viscosity, the diffusion rate of molecules may be reduced, potentially impairing the detection efficiency of biosensors that rely on diffusion processes. This is particularly critical in scenarios where rapid contact between target molecules and the biosensor surface is essential for detection. Furthermore, high viscosity might compromise the uniformity of contact between the liquid biopsy solution and the biosensor surface, subsequently diminishing the efficiency of biosensors in capturing target molecules.^1^ Therefore, viscous samples must be diluted with phosphate-buffered saline (PBS) or lysis buffer before detection, which may affect the efficiency of biomarker concentration measurement. The clinical liquid biopsies available for testing include blood, serum, plasma, urine, tears, interstitial fluid (ISF), saliva, and sputum. Among these, skin ISF is an especially rich source of soluble biomarkers, such as proteins, peptides, metabolites, and nucleic acids, which show a close correlation with blood.^2−5^ However, extraction of ISF followed by *ex vivo* analysis has not been widely embraced in preclinical or clinical applications. This is mainly due to the difficulties associated with ISF extraction, which is time-consuming and requires bulky instruments, making comprehensive analysis challenging. In contrast, saliva and sputum represent relatively easy-to-obtain and noninvasive liquid biopsies. Salivary biomolecules can serve as reliable diagnostic tools for detecting various respiratory infections (e.g., Influenza A and COVID-19), malignancies (e.g., head and neck squamous cell carcinoma), genetic diseases, and hormonal abnormalities.^6−8^ However, their higher viscosity compared to other liquid biopsies, along with interindividual variability, may affect biosensors’ detection sensitivity and accuracy. Additionally, not all disease biomarkers are present in saliva and sputum. Therefore, it is crucial to design a biosensor capable of effectively capturing target protein biomarkers in both epidermis and liquid biopsies, particularly those with high viscosity.

In recent decades, much attention has been directed at using microneedle patches (MNP) for minimally invasive transdermal drug and vaccine delivery. Compared to hypodermic needles, MNP avoid the nerves and vascular structures located in the deeper layers of the dermis, thereby significantly minimizing their associated pain and risks of infection.^9^ Recently, the utility of MNP for minimally invasive transdermal biosensing has also been reported and attracted significant attention. In general, MNP-based biosensing tools can be classified into three main categories according to their application purposes: (1) MNP is designed for extraction of ISF or capture of biomarkers selectively followed by *ex vivo* analysis by external methods;^10−12^ (2) MNP is integrated into a sensor to detect or monitor the analytes within the skin;^13,14^ and (3) MNP is used for biosignal recording platforms.^15,16^ However, MNP-assisted extraction yields a small amount of ISF (approximately 2–3 μL of ISF from 4 cm^2^ of human skin), which is insufficient for comprehensive proteomic and metabolomic analysis.^17^ Unlike ISF extraction, MNP functionalized with biorecognition elements (e.g., antibodies, antigens, aptamers) can directly and specifically capture target biomarkers within epidermal tissues, followed by on-needle analysis.^18,19^ This approach presents a promising avenue for straightforward and effective biodetection.^20,21^ Nevertheless, friction during skin penetration may compromise the adhesion of biorecognition elements coated or immobilized on the surface of MNP, leading to a reduction in biomarker capture efficiency. The concentrations of biomarkers in ISF are generally lower than those found in blood. Furthermore, not all disease biomarkers are present in ISF, particularly in the context of respiratory viral infections.^3^ Additionally, the binding kinetics between analytes and biorecognition elements are adversely affected by the dense tissue environment, which impedes the diffusion of target biomolecules toward the surface of MNP. This phenomenon reduces the likelihood of analyte capture and subsequently diminishes the signal intensity associated with the analyte.

Here, we have designed and fabricated an MNP-based sensing box, termed MNP-based SenBox, for mobile healthcare testing (MHCT). The objective is to achieve ultrasensitive and quantitative detection of protein biomarkers in the epidermal tissues or liquid biopsies, particularly in viscous samples such as saliva and sputum, followed by on-needle analysis for immediate assessment. The incorporation of horseradish peroxidase-incorporated zeolitic imidazolate framework-8 (HRP@ZIF-8) as a sensitive and stable signal probe enhances the improvement of the limit of detection of various target protein biomarkers and significantly accelerates the biomarker detection process. Moreover, by employing a layer-by-layer (LBL) design of MNP, we successfully achieved uniform deposition of gold nanoparticles (AuNPs) on MNP to facilitate the self-assembly of antigens or antibodies on MNP. Using clinical saliva and sputum samples combined with an inflammation rat model, we demonstrated that the MNP-based SenBox enables ultrasensitive and quantitative monitoring of various protein biomarkers through a straightforward stick-and-peel procedure followed by on-needle analysis. First, we demonstrated on-needle detection of anti-SARS-CoV-2 N-protein (NP) IgA antibodies and S1 proteins (S1P) from SARS-CoV-2 strains in viscous saliva/sputum to enable precise and early detection of viral infections. Second, we validated the application of MNP-based SenBox in detecting and quantifying inflammatory biomarker levels in rats after induction of Complete Freund’s Adjuvant (CFA)-induced inflammation. The MNP-based SenBox can perform noninvasive and minimally invasive biosensing, thereby obviating the need for target tissue destruction and repeated blood draws over a short period, which can improve patient compliance with regular follow-up testing. This highly sensitive biosensing technology has the potential for extensive application of MNP-based SenBox in the capture and analysis of protein biomarkers in epidermal tissues or liquid biopsies, particularly those with high viscosity.

## Results/Discussion

### Design and Fabrication of the MNP-Based SenBox

**Figure 1:**
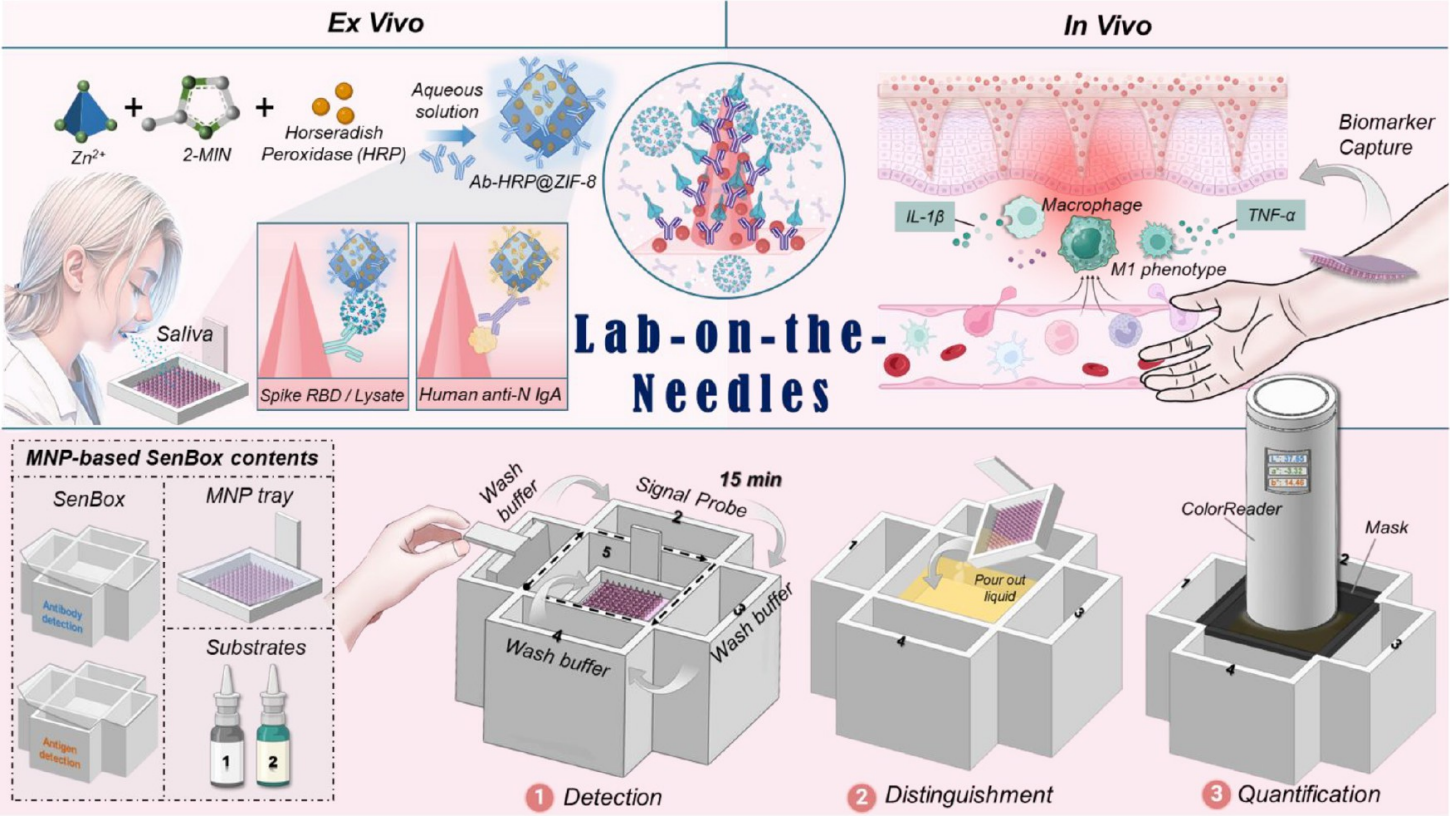
Schematic illustration of the MNP-based SenBox for rapid, mobile healthcare protein biomarker detection in epidermis and liquid biopsies, particularly those with high viscosity. It showcases the comprehensive procedures of the MNP-based SenBox platform, which integrates with a portable colorimetric analysis system.

**Figure 2:**
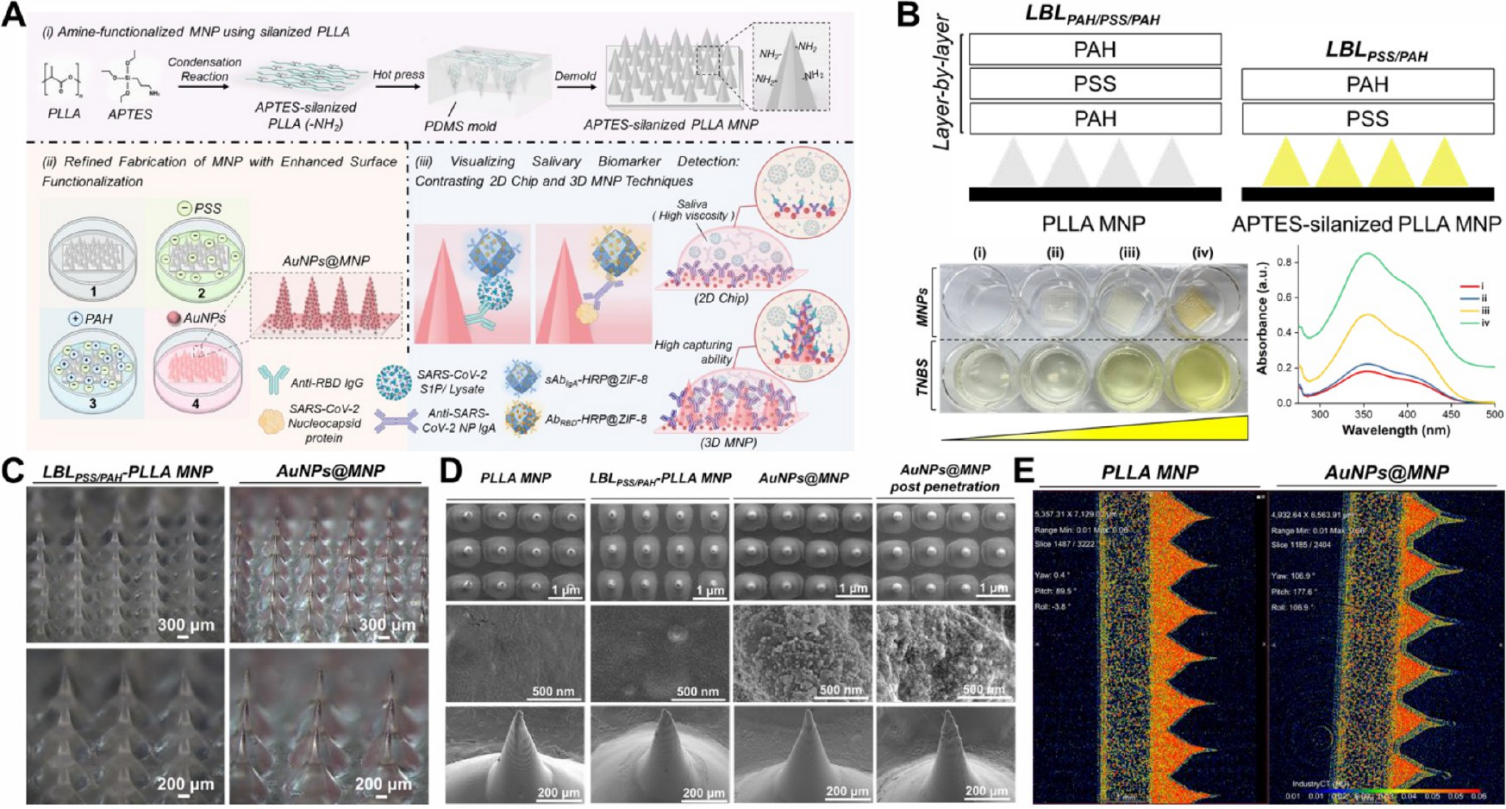
(A) It outlines the developmental journey and deteecting mechanism of the MNP-based rapid test, detailing the preparation processes of (i) APTES-silanized PLLA MNP, (ii) AuNPs@MNP, and (iii) Ab_RBD_@MNP/NP@MNP. (B) Schematic representation of the LBL process for LBL_PAH/PSS/PAH_-PLLA MNP and LBL_PSS/PAH_-PLLA MNP. Qualitative analysis of the presence of amine groups (−NH_2_) on (ii) PLLA MNP, (iii) LBL_PAH/PSS/PAH_-PLLA MNP, and (iv) LBL_PSS/PAH_-PLLA MNP through TNBS staining, compared with (i) TNBS solution only. (C) Bright-field images of LBL_PSS/PAH_-PLLA MNP before (left) and after (right) a 30 min exposure to AuNPs (AuNPs@MNP). (D) Representative SEM images of PLLA MNP, LBL_PSS/PAH_-PLLA MNP, AuNPs@MNP, and AuNPs@MNP post penetration. Upper: top view; middle: partial enlargement; lower: side view. (E) MicroCT images of LBL_PSS/PAH_-PLLA MNP and AuNPs@MNP.

The biosensing technology introduced in this study relies on MNP functionalized with biorecognition elements (such as antibodies or antigens) that selectively capture protein biomarkers (such as antigens or antibodies) in epidermal tissues or liquid biopsy samples in a concentration-dependent manner combined with the newly designed operation box. Subsequently, the protein biomarkers captured on the surface of MNP are quantified by an ultrasensitive colorimetric immunoassay using a portable ColorReader (Figure 1 and Video S1). For efficient capture of protein biomarkers in epidermal tissues and viscous liquid biopsies, MNP is required to exhibit high biorecognition elements conjugation efficiency and protein–antibody binding ability. Thus, we coated gold nanoparticles (AuNPs) on the surface of MNP in a self-assembled manner to make the surface rougher, increase the surface area, and simplify the biorecognition element conjugation process. To achieve this goal, we first set out to create MNP with amine-rich surfaces by assembling polyelectrolyte multilayers (PEMs), nanostructured films formed by iterative layer-by-layer (LBL) adsorption of poly(sodium-4-styrenesulfonate) (PSS) and poly(allylamine) hydrochloride (PAH) on 3-aminopropyltriethoxysilane (APTES)-silanized poly(l-lactide) (PLLA) MNP, resulting in LBL_PSS/PAH_-PLLA MNPs (Figure 2A). In the Fourier transform infrared (FTIR) spectra depicted in Figure S1, the presence of N–H stretching vibration is noted at 3230 cm^–1^. At the same time, absorption peaks are observed at 1082 and 1267 cm^–1^, indicating the presence of Si–O–Si and Si–O–C bonds, respectively,^22^ in APTES-silanized PLLA MNP. These findings provide evidence that PLLA MNP was indeed covalently functionalized with APTES. Subsequently, the 2,4,6-trinitrobenzenesulfonic acid (TNBS) assay^23^ was used to confirm the successful amine group coating on the surface of LBL_PSS/PAH_-PLLA MNP. As illustrated in Figure 2B, the LBL_PSS/PAH_-PLLA MNP group exhibited a deeper yellow color and higher absorbance intensity at 345 nm compared to both naked PLLA MNP and LBL_PAH/PSS/PAH_-PLLA MNP. This observation suggests that pretreatment of PLLA MNP with APTES promotes the formation of PEMs on the MNP surface, resulting in a strong positive charge that facilitates the subsequent self-assembly of negatively charged AuNPs (−22.5 ± 2.8 mV) with a uniform size of 20.9 ± 1.5 nm on the MNP surface (Figure S2A). Then, we investigated the optimal self-assembly time of AuNPs on the MNP’s surface by monitoring the absorbance intensity at 530 nm using a UV–vis-NIR spectrometer (MODEL V-700, JASCO, Japan). The results demonstrated that the absorption peak attributed to AuNPs at 530 nm is detectable only 0.5 h after the initiation of the self-assembly process. It reaches a stable level within 6 h. With prolonged time, not only does the absorption intensity at 530 nm fail to increase further, but the phenomenon of AuNPs aggregation and stacking emerges (Figure S2B). Consequently, the optimal duration for AuNPs to self-assemble and form a thin layer on the surface of LBL_PSS/PAH_-PLLA MNPs (AuNPs@MNP) is 0.5 to 6 h. As shown in Figure 2C via optical microscopy, the LBL_PSS/PAH_-PLLA MNP exhibits a conical morphology characterized by a length of approximately 1,000 μm, a base width of approximately 890 μm, a sharp tip measuring around 500 μm, and an inter-MN spacing of 800 μm. The claret coloration observed on the AuNPs@MNP suggests the successful self-assembly of the AuNPs layer on the LBL_PSS/PAH_-PLLA MNP surface. Scanning electron microscopy (SEM) images reveal numerous small particles attributed to AuNPs, as confirmed by energy-dispersive X-ray spectroscopy (EDS) analysis (Figure S3). These results indicate a uniform deposition of AuNPs on the MNP surface in the AuNPs@MNP group compared to the LBL_PSS/PAH_-PLLA MNP group. Furthermore, the AuNPs deposited on the surface of the microneedles were not significantly dislodged after penetration into rat skin, along with some adhered tissue mucosa, as confirmed by SEM (Figure 2D). This ensures sufficient antibodies were on the microneedles to capture protein biomarkers in the skin ISF. The microcomputed tomography (microCT) images depict the heterogeneity of the PLLA MNP structure, with the color coding representing relatively high density (red), medium density (yellow-green), and low density (blue). The results demonstrate that the uniformly thin layer of AuNPs on the surface of LBL_PSS/PAH_-PLLA MNP influences its X-ray absorption characteristics in microCT imaging. This reduces bright regions compared to LBL_PSS/PAH_-PLLA MNP, indicating a uniformly thin layer of AuNPs covering the entire MNP, including both the base patch and needles (Figure 2E). This significantly enhances the surface area for conjugating more biorecognition elements and facilitates the capture of more protein biomarkers that may be challenging to diffuse in viscous samples. The findings mentioned above suggest successful coverage of the AuNPs layer onto the LBL_PSS/PAH_-PLLA MNP, facilitating subsequent biorecognition elements (could be antigen or antibody) conjugation. Furthermore, we investigated the optimal duration for AuNPs coating to facilitate Ab_RBD_ conjugation on AuNPs@MNP. The results showed that peak binding efficiency was achieved in just 0.5 h. This suggests that a mere 0.5-h self-assembly time of AuNPs on LBL_PSS/PAH_-PLLA MNP is adequate for biorecognition element modification on AuNPs@MNP to reach saturation. This will significantly shorten the preparation time for MNP-based biosensors (Figure S4A). Subsequently, localized surface plasmon resonance (LSPR) analysis was employed to verify the conjugation of Ab_RBD_ onto AuNPs@MNP via Au–S bonding. LSPR is highly sensitive to changes in refractive index, inducing a shift in the LSPR position upon conjugation of molecules to the surface of metallic nanoparticles.^24,25^ Following Ab_RBD_ conjugation, a noticeable plasmon red shift in the LSPR peak was observed in the spectrum. This indicated a 6 nm spectral redshift after Ab_RBD_ conjugation and a further 9 nm spectral redshift after BSA blocking (Figure S4B), confirming the successful preparation of Ab_RBD_@MNP.

To facilitate antibody–antigen interactions, optimized concentrations of thiol-modified SARS-CoV-2 N-protein (SH-NP) and thiol-modified anti-RBD IgG antibodies (SH-Ab_RBD_) were self-assembled onto AuNPs@MNP, resulting in NP@MNP for detecting anti-SARS-CoV-2 NP IgA antibody and Ab_RBD_@MNP for detecting SARS-CoV-2 S1 protein (SARS-CoV-2 S1P). The successful immobilization of Ab_RBD_ and its efficiency were also validated between the AuNPs-coated two-dimensional (2D) LBL_PSS/PAH_-PLLA chip (AuNPs@chip) and AuNPs@MNP using enzyme-linked immunosorbent assay (ELISA). Up to 249.2 ± 5.8 ng of Ab_RBD_ could be bound to the AuNPs@MNP with a conjugation rate of 99.7 ± 2.3% for 1 h of incubation. Additionally, the SH-Ab_RBD_ was immobilized on the AuNPs@chip to form Ab_RBD_@chip using the same parameters. Notably, only 168.7 ± 4.5 ng of Ab_RBD_ could be bound to AuNPs@chip to form Ab_RBD_@chip, with a decreased conjugation rate of 32.5 ± 1.8% compared to Ab_RBD_@MNP (Figure S5). The results showed that AuNPs@MNP, with its three-dimensional (3D) structure, possesses a larger surface area for modifying more biorecognition elements, potentially enhancing the protein capture rate in epidermal tissues and liquid biopsy samples, particularly in viscous saliva. However, the distribution uniformity of biorecognition elements (antibodies or antigens) conjugated on the MNP will significantly influence the capture efficiency of target proteins, especially for *in vivo* skin ISF protein detection. If the needle tip lacks sufficient antibodies, it may not effectively capture proteins in the skin ISF, reducing detection sensitivity. To confirm the uniform distribution of biorecognition elements on the MNP, we conjugated ABflo 647-labeled rabbit anti-rat IgG antibody (Ab_IgG_) onto the MNP, forming Ab_IgG_@MNP, followed by staining with ABflo 647-labeled goat anti-rabbit IgG antibody. The results showed uniform red fluorescence across the MNP surface, indicating that the antibodies were evenly distributed across the entire surface of the MNP, from the needle tips to the funnel bases, rather than being sporadically localized (Figure S6).

Additionally, the long-term stability and durability of the biorecognition elements (antibodies or antigens) conjugated onto MNP are crucial factors for evaluating the long-term storage of MNP-based SenBox. The prepared NP@MNP and Ab_IL-1β_@MNP samples were stored under different temperature conditions (25, 37, and 50 °C) for 3, 6, 9, 12, 15, 18, and 21 days. As shown in Figure S7, the detection efficiency of NP@MNP and Ab_IL-1β_@MNP decreased by only 15% after 21 days of storage at 50 °C. Notably, the prepared NP@MNP and Ab_IL-1β_@MNP exhibited no loss in detection efficiency when stored at 25 °C for 21 days compared to the initial detection efficiency (day 0). This excellent stability is likely due to the biorecognition elements conjugated onto MNP via covalent bonds, which limit their degree of freedom, thus reducing conformational changes and enhancing stability under varying temperature conditions.^26^

### Synthesis and Characterization of the Highly Sensitive HRP@ZIF-8

**Figure 3:**
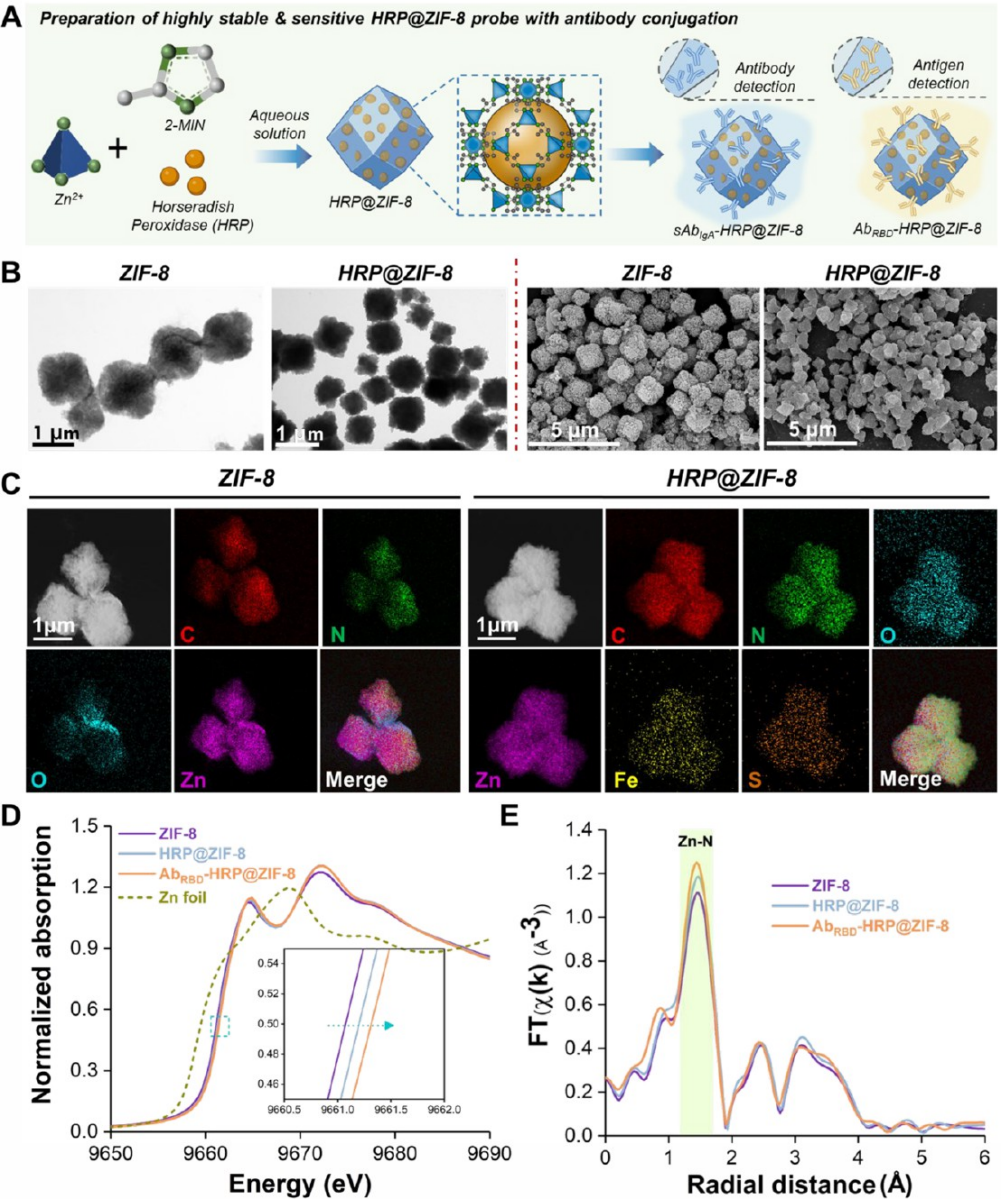
(A) A synthetic illustration depicts the highly stable HRP@ZIF-8 signal probes conjugated with specific antibodies. (B) TEM (left) and SEM (right) images of ZIF-8 and HRP@ZIF-8 nanoparticles synthesized through the bulk solution. (C) EDS element mapping images of the ZIF-8 and HRP@ZIF-8 nanoparticles. (D) XANES spectra at the Zn K-edge of ZIF-8, HRP@ZIF-8, Ab_RBD_-HRP@ZIF-8, and Zn foil. Inset: magnification of the shift in absorption edge energy at half-height. (E) Fourier transforms of Zn K-edge EXAFS spectra of ZIF-8, HRP@ZIF-8, and Ab_RBD_-HRP@ZIF-8.

In addressing the limitations associated with current detection signal sources for MHCT, it is essential to reduce the operational complexity inherent in conventional ELISA procedures while enhancing detection sensitivity and signal source stability. The ZIF-8 emerges as a crucial material due to its capacity to encapsulate many enzymes and stabilize their structure. Additionally, ZIF-8 facilitates rapid covalent bonding with thiolated biomolecules, thus serving as a scaffold for signal probes. Hence, a one-step process was utilized to produce HRP@ZIF-8 at room temperature as the scaffold for the high-sensitivity detection signal probe essential for this study (Figure 3A). From transmission electron microscopy (TEM) and SEM images (Figure 3B), ZIF-8 and HRP@ZIF-8 exhibit the characteristic rhombic dodecahedron morphology typical of ZIF-8.^27^ The synthesized ZIF-8, serving as the control, has a diameter of 1.5 μm with a porous structure. At the same time, HRP@ZIF-8 appears as smaller particles (800 nm in diameter, 46% size reduction) with a partially solid morphology,^28^ likely due to the presence of multiple nucleation seeds (heterogeneous nucleation process), leading to a more significant number of smaller crystals and a denser structure. The heterogeneity in particle size is attributed to the varying degrees of HRP loading within individual ZIF-8 particles. Despite this size variation, it does not adversely affect catalytic performance, as the encapsulation process creates a favorable microenvironment that stabilizes and enhances enzyme activity. The structural integrity of both ZIF-8 and HRP@ZIF-8 was confirmed through X-ray diffraction (XRD) analysis (Figure S8). The presence of strong peaks at Bragg angles (2θ) = 7.3°, 10.4°, 12.7°, 14.7°, 16.5°, and 18.0° correspond to Miller indexes for planes of (011), (002), (112), (022), (013), and (222), respectively, which indicates high crystallinity of the prepared ZIF-8 with rhombic dodecahedron morphology. The prepared HRP@ZIF-8 shows similar peaks with virgin ZIF-8, indicating their crystal structure remains unchanged after introducing HRP encapsulation. The EDX mapping of HRP@ZIF-8 shown in Figure 3C, demonstrates the presence of elemental Zn, C, N, and O, which are anticipated constituents of ZIF-8. Additionally, the detection of elemental Fe and S is attributed to the presence of HRP. These findings confirm the successful encapsulation of HRP within the ZIF-8 framework. The thermal gravimetric analysis (TGA) curves of ZIF-8 and HRP@ZIF-8 are shown in Figure S9. In comparison to ZIF-8, the initial weight loss of 17.3 wt % in HRP@ZIF-8 between 150 and 380 °C is attributed to the degradation of the incorporated HRP, indicating a loading ratio of approximately 17.3 wt % for HRP in HRP@ZIF-8 (approximately 0.173 μg HRP/μg HRP@ZIF-8). The subsequent weight loss of HRP@ZIF-8 at temperatures higher than 380 °C results from the degradation of ZIF-8 frameworks. These results collectively confirm the successful encapsulation of HRP within ZIF-8. The Brunauer–Emmett–Teller (BET) analysis was performed to assess changes in the specific surface area and pore size of ZIF-8 following HRP encapsulation. The results indicated a reduction in pore size from 2.19 to 1.66 nm (approximately 24% decrease) and a reduction of specific surface area from 763.02 m^2^/g to 738.26 m^2^/g (approximately 3% decrease), demonstrating the successful encapsulation of HRP within the ZIF-8 pores. Moreover, the reduction in pore size likely influences molecules’ diffusion and transport behavior, restricting the entry of larger molecules while improving selectivity for smaller ones (Figure S10). Despite the decrease, the pore size remains suitable for exposing the catalytic active sites of HRP and facilitating the efficient penetration of the colorimetric substrate into HRP@ZIF-8, thereby promoting catalytic reactions and generating a significant yellow color signal (Figure S11). To further verify the encapsulation of HRP within ZIF-8 pores, we conjugated thiolated HRP (HRP_SH_) to the surface of HRP@ZIF-8, resulting in HRP_SH_-HRP@ZIF-8. We then measured the catalytic activity of HRP@ZIF-8 and HRP_SH_-HRP@ZIF-8 before and after trypsin treatment by reacting them with TMB/H_2_O_2_. The results showed that the activity of HRP_SH_-HRP@ZIF-8 returned to a level similar to that of HRP@ZIF-8, indicating that the surface-conjugated HRP was degraded by trypsin, leaving only the HRP encapsulated within ZIF-8 (Figure S12). These findings confirm that HRP in the prepared HRP@ZIF-8 is indeed encapsulated within the pores of ZIF-8 rather than being adsorbed on the surface.

To elucidate the effect of the zinc (Zn) site of ZIF-8 on HRP and anti-SARS-CoV-2 RBD antibody (Ab_RBD_), the chemical state and local structure of Zn was conducted for ZIF-8, HRP@ZIF-8, and Ab_RBD_-HRP@ZIF-8 as well as a reference of Zn foil. In X-ray Absorption Near Edge Structure (XANES) spectroscopy, the intense feature of ZIF-8, resulting from a dipole transition from 1s to 4p located to the right of Zn foil, indicates the presence of a positively charged Zn atom in the ZIF-8. The oxidation state of the Zn site increased upon incorporation of HRP into the ZIF-8 cage. This state further increased when the Ab_RBD_ was conjugated to the surface of the ZIF-8 cage, indicating a redistribution of the electronic structure of Zn (Figure 3D). Meanwhile, the Fourier-transformed (FT) k3-weighted extended X-ray absorption fine structure (EXAFS) spectra of the ZIF-8 exhibit a main peak at around 1.46 Å, resulting from a single scattering by the nearest neighboring N atom. In HRP@ZIF-8, the findings reveal a similar structure characterized by intense scattering of Zn–N bonds at 1.46 Å. However, there is a notable increase in coordination number, suggesting the formation of a ligand between Zn and HRP. The observed ligand is likely the imidazole structure within the HRP, coordinating with Zn in the ZIF-8. This ligand likely facilitates the dispersion of electron density associated with the Zn atom, consequently resulting in an elevated oxidation state. This correlation aligns with the findings from XANES results (Figure 3E). The coordination number increases further as the Ab_RBD_ is incorporated into HRP@ZIF-8, implying the formation of an additional ligand. Notably, the atomic radius of Zn is reduced due to the increasing oxidation state of the Zn atom, resulting in a slight shortening of bond lengths. Changes in the electronic and local structure reveal that the Zn site in ZIF-8 plays a critical binding role. This allows for the secure encapsulation of HRP within the ZIF-8 cage and the immobilization of the Ab_RBD_ on the ZIF-8 surface, thereby stabilizing their configuration and activity even in harsh environments (such as extreme pH, elevated temperature, and the presence of proteases). Most relevant literature has indicated that the peroxidation activity of HRP in the TMB/H_2_O_2_ oxidation reaction is susceptible to disruption by the pH of the surrounding environment; however, it may not suit all biomolecules for detection. The findings in Figure S13A demonstrate the sensitivity of HRP peroxidation activity to pH, with optimal performance observed at pH 6. The activity gradually decreased when the pH was higher or lower 6, potentially attributed to the induced unfolding of HRP into a disordered polypeptide chain, leading to the misalignment of amino acid residues crucial for enzyme function interactions^29^ in more alkaline or acidic environments. Conversely, the peroxidase activity of HRP@ZIF-8 remained unaffected by pH variations, effectively oxidizing TMB/H_2_O_2_ and maintaining consistent signal generation across a broad pH range from 3 to 11. Compared to previous reports indicating that the structure of ZIF-8 loses coordination under acidic conditions or in PBS,^30^ our prepared polyvinylpyrrolidone (PVP)-assisted ZIF-8, coated with poly(vinyl alcohol) (PVA) and bovine serum albumin (BSA), was found to retain its protective capability under extreme conditions for a longer period. This is likely due to (1) the ability of PVP to coordinate with zinc atoms in ZIF-8 nodes and adsorb 2-methylimidazole (2-MIM) linkers via hydrophobic interactions, thereby strengthening the ZIF-8 structure and (2) the PVA and BSA coating providing a physical barrier to chelating agents (e.g., PO_4_^3–^ ligands) that could destabilize the ZIF-8 framework, thereby reducing protonation and subsequent breakdown of the metal–ligand bonds within the ZIF-8 framework, which enhances colloidal stability.^31,32^ To verify this, we analyzed the catalytic activity of stored Ab_RBD_-HRP@ZIF-8 in PBS at 4 °C various time points (t = 1, 2, 3, 4, 7, 15, 30, 60, and 120 days). The results showed that theabsorbance intensity at 450 nm (*A*_450nm_) values obtained from purified Ab_RBD_-HRP@ZIF-8 at each time point exhibited no significant difference compared to the initial *A*_450nm_ (t = 0), indicating that Ab_RBD_-HRP@ZIF-8 maintained its intact structure and did not decompose in PBS to release the encapsulated HRP (Figure S14). Significantly, in the presence of trypsin, the HRP@ZIF-8 essentially retained all of the initial peroxidation activity of HRP after 240 min of incubation. In contrast, the nonprotected HRP exposed to trypsin completely lost its original activity (Figure S13B). Furthermore, HRP@ZIF-8 also exhibits exceptional thermal stability. As depicted in Figure S13C, naked HRP exhibited a complete activity loss after 2 days of storage at 50 °C. Similarly, complete peroxidation activity loss was observed after 21 days of storage at 25 °C. However, the HRP@ZIF-8 was used to oxidize TMB/H_2_O_2_ without any loss in sensitivity, even when stored at 25 and 37 °C for 21 days. Impressively, only 42.4% of the initial peroxidation activity of HRP@ZIF-8 was lost after 21 days of storage at 50 °C. The above experiments confirm that the ZIF-8 coating acts as a protective layer for the enzyme and the formation of stronger bonds (i.e., hydrogen bonds and van der Waals forces) between HRP and ZIF-8 that allows diffusion of the substrate (TMB/H_2_O_2_) through the ZIF-8 pore cavities while preventing the ingress of the proteolytic agent trypsin and also stabilize the configuration of HRP.^33−35^ Further exploration into the enzymatic kinetics of the HRP showed that the Michaelis–Menten constant, *K*_*m*_, of the free HRP was 122.2 μM. In contrast, for encapsulated HRP (HRP@ZIF-8), *K*_*m*_ was increased to 217.1 μM. This increase is likely due to the porous structure of ZIF-8, which may cause the substrate to take longer to diffuse to the active site of the encapsulated HRP. However, once the substrate reaches the active site, the catalytic efficiency is enhanced, resulting in a higher *V*_*max*_ (increased to 3.1 μM/s from 1.2 μM/s) due to increased stability and an optimized microenvironment that reduces product feedback inhibition (Figure S15).^36,37^ The aforementioned results corroborate that our method of synthesizing HRP@ZIF-8 at ambient temperature significantly enhances the stability of HRP while augmenting its catalytic efficiency.

Subsequently, the conjugated efficiency of recognized antibodies on HRP@ZIF-8 was investigated. The thiolated antihuman IgA secondary antibody (sAb_IgA_) or Ab_RBD_ was bound to HRP@ZIF-8 through Zn–S bonds to form sAb_IgA_-HRP@ZIF-8 as a signal probe for anti-SARS-CoV-2 NP IgA antibody detection or Ab_RBD_-HRP@ZIF-8 as a signal probe for SARS-CoV-2 S1P detection. Approximately 97.9 ± 0.2 ng of 100 ng sAb_IgA_ was bound onto 677 μg HRP@ZIF-8 with a conjugation efficiency of 0.145 ng sAb_IgA_/μg HRP@ZIF-8 (Figure S16A, C). Approximately 97.6 ± 0.1 ng of 100 ng Ab_RBD_ was bound onto 677 μg HRP@ZIF-8 with a conjugation efficiency of 0.144 ng Ab_RBD_/μg HRP@ZIF-8 (Figure S16B, D).

### *ExVivo* Detection of COVID-19-Related Proteins in Saliva

**Figure 4:**
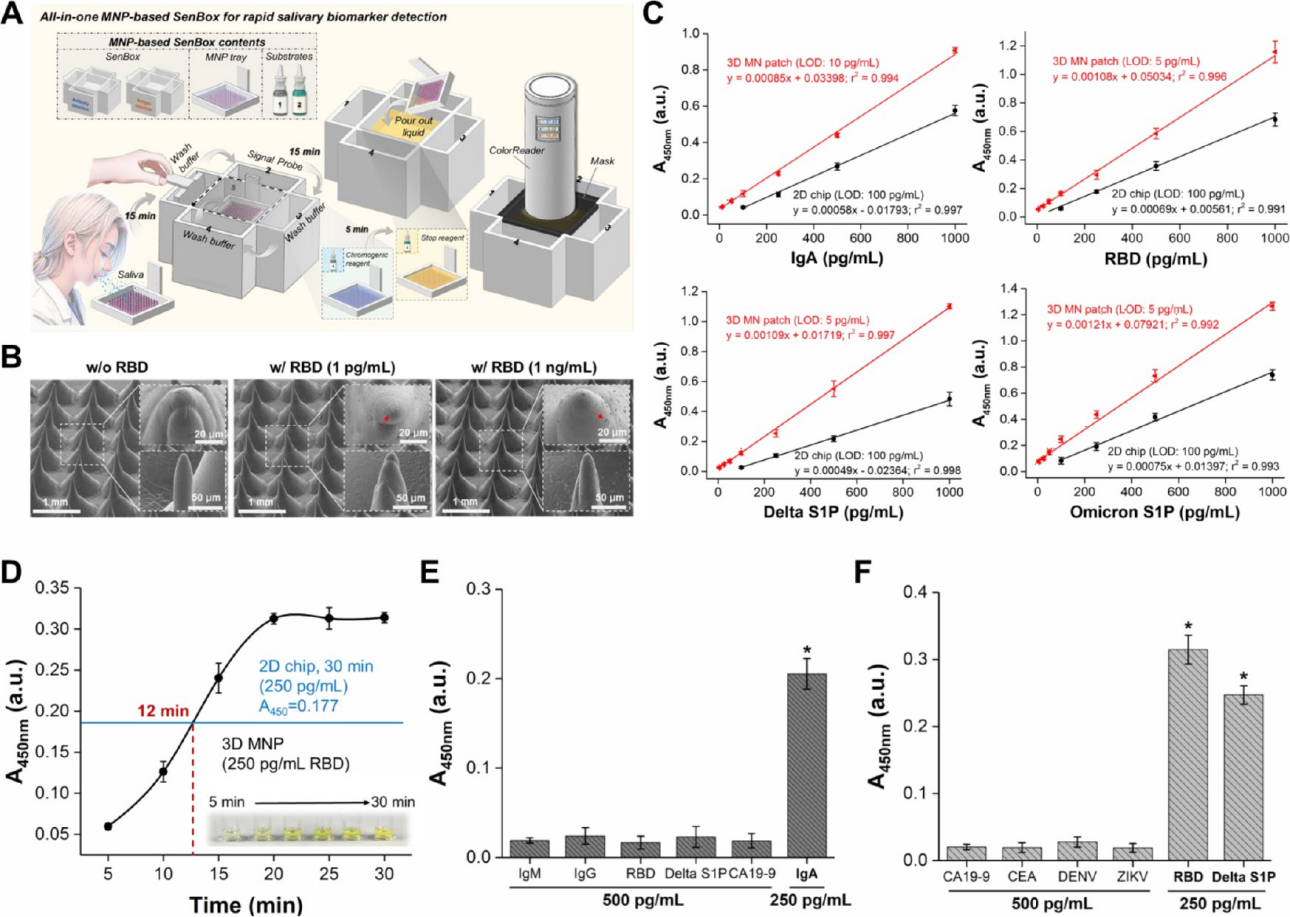
(A) Schematic illustration of the MNP-based SenBox integrated with a portable ColorReader for rapid, point-of-care protein biomarker detection in viscous saliva. (B) SEM images of Ab_RBD_@MNP incubated with Ab_RBD_-HRP@ZIF-8 in the absence and presence of low concentration (1 pg/mL) of RBD and high concentration (1 ng/mL) of RBD. The small particles indicated by the red arrow represent the Ab_RBD_-HRP@ZIF-8 signal probes that remained on the Ab_RBD_@MNP in the presence of RBD. (C) Calibration curves for various biomarkers (IgA antibody, RBD, Delta S1P, Omicron S1P) were plotted over a range of concentrations (5 pg/mL to 1,000 pg/mL) using spectrophotometric measurements at 450 nm (*n* = 3). (D) Comparison of time-dependent signal intensity in immunobinding tests with 250 pg/mL RBD protein spiked in viscous salvia using 2D Ab_RBD_@chip and 3D Ab_RBD_@MNP (*n* = 3). The inset displays the time-dependent color changes of the detection results using 3D Ab_RBD_@MNP. (E) Anti-interference test results using NP@MNP for specific detection of IgA (250 pg/mL) amidst various interfering substances (500 pg/mL) (*n* = 3). *indicates a significant difference (Student’s *t*-test, *p ≤ 0.05). (F) Anti-interference test results using Ab_RBD_@MNP for specific detection of RBD protein (250 pg/mL) and Delta S1P (250 pg/mL) amidst various interfering substances (500 pg/mL) (*n* = 3). *indicates a significant difference (Student’s *t*-test, *p ≤ 0.05). ^#^ IgA: anti-SARS-CoV-2 NP IgA antibody; IgM: anti-SARS-CoV-2 NP IgM antibody; IgG: anti-SARS-CoV-2 NP IgG antibody; Delta S1P: SARS-CoV-2 Delta S1P; Omicron S1P: SARS-CoV-2 Omicron S1P.

In liquid biopsy samples with elevated viscosity, the diffusion rate of molecules may decrease, potentially affecting the uniformity of contact between the liquid biopsy solution and the biosensor surface. This phenomenon can subsequently influence the efficiency of the biosensor in capturing target biomarkers. Thus, we introduce an MNP-based SenBox for the ultrasensitive, convenient, and rapid detection of target biomarkers in viscous liquid biopsy (Figure 4A). The results presented in Figure 4B demonstrate that no Ab_RBD_-HRP@ZIF-8 remained on Ab_RBD_@MNP after incubation with Ab_RBD_-HRP@ZIF-8 without recombinant RBD. Conversely, a notable quantity of Ab_RBD_-HRP@ZIF-8 was observed on Ab_RBD_@MNP in the presence of recombinant RBD at a concentration of 1 ng/mL. Even at concentrations of RBD as low as 1 pg/mL, traces of Ab_RBD_-HRP@ZIF-8 were still observed on the Ab_RBD_@MNP.

While precise quantification of anti-SARS-CoV-2 NP IgA antibody and SARS-CoV-2 S1P in saliva post-SARS-CoV-2 infection is not essential in COVID-19 rapid testing, comprehension of their concentrations can provide additional insights into the infection stage for patients. Hence, we assessed the detection sensitivity and linear detection range of MNP-based SenBox for anti-SARS-CoV-2 NP IgA antibody, RBD, SARS-CoV-2 Delta S1P, and SARS-CoV-2 Omicron S1P (Figure 4C). The standard curve of the 2D chip demonstrated linearity within the 100 pg/mL to 1,000 pg/mL range (r^2^ = 0.997) for detecting anti-SARS-CoV-2 NP IgA antibody using sAb_IgA_-HRP@ZIF-8 as the signal probe. Compared to the 2D chip, the detection sensitivity of the 3D NP@MNP increased by 10-fold, and its limit of detection (LOD) was reduced to 10 pg/mL from 100 pg/mL. Similar detection trends were observed in detecting RBD, Delta S1P, and Omicron S1P. Compared to the 2D chip, the detection sensitivities of the 3D Ab_RBD_@MNP for RBD, SARS-CoV-2 Delta S1P, and SARS-CoV-2 Omicron S1P were all improved by 20-fold, with their respective LOD all reduced to 5 pg/mL from 100 pg/mL. The results demonstrate that the 3D Ab_RBD_@MNP indeed possesses a higher efficiency in molecular capture compared to the 2D chip. This outstanding characteristic is particularly evident in samples with higher viscosity, where its advantages are more pronounced. To substantiate this claim, the 2D chip was subjected to incubation with viscous saliva spiked with 250 pg/mL RBD for a duration of 30 min, yielding a measured *A*_450nm_ of 0.177. Following this, we exposed the 3D Ab_RBD_@MNP to viscous saliva containing same concentration of RBD and monitored the *A*_450nm_ values at 5 min intervals. The findings indicated that it took only 12 min for the *A*_450nm_ to reach 0.177, demonstrating a significant improvement in detection sensitivity. This enhancement is primarily attributed to the viscous saliva droplet behaving similarly to a thin soft tissue with an approximate thickness of 2 mm. The needles of Ab_RBD_@MNP can penetrate the droplet, efficiently capturing the RBD molecules both within the interior and near the interface between the base of MNP and the droplet. In contrast, the 2D chip is capable of capturing RBD molecules located near the interface between the 2D chip and the droplet but is unable to capture the slow-moving RBD molecules dispersed within the interior of the droplet (Figure 4D). The results confirm that the MNP-based SenBox exhibits outstanding efficacy in detecting protein biomarkers across all types of liquid biopsies, particularly those with high viscosity.

Following the initial characterization, we conducted specificity assessments of the MNP-based SenBox by incubating it with samples spiked with various interfering molecules. These assessments confirmed that the interfering molecules did not affect the detection and quantification of the target protein biomarkers (anti-SARS-CoV-2 NP IgA antibody and various mutant strains of SARS-CoV-2 S1P) by the MNP-based SenBox (Figure 4E and 4F). This indicates that the MNP-based SenBox can be used for efficient *ex vivo* on-needle detection of protein biomarkers in liquid biopsies, exhibiting high sensitivity and specificity. To validate the accuracy of the MNP-based SenBox in detecting the anti-SARS-CoV-2 NPIgA antibodies, RBD, Delta S1P, and Omicron S1P, we introduced various concentrations of anti-SARS-CoV-2 NP IgA antibody, RBD, or SARS-CoV-2 Delta S1P into human saliva, ranging from 50 pg/mL to 1,000 pg/mL, and subsequently recorded the corresponding *A*_450nm_ values using a microplate spectrophotometer; the results are summarized in Table 1. The observed recovery rates were deemed satisfactory, falling within the range of 99.3–104.5% for anti-SARS-CoV-2 NP IgA antibody detection, 99.1–103.2% for RBD detection, and 100.6–104.8% for SARS-CoV-2 Delta S1P detection, with all relative standard deviation (RSD) values remaining below 5%. To demonstrate that the MNP-based SenBox can be used for accurate protein detection across a broader range of liquid biopsies, we spiked various concentrations of anti-SARS-CoV-2 NP IgA antibody, RBD, or SARS-CoV-2 Delta S1P into PVA solutions (ranging from 1 wt % to 10 wt %, with viscosities from 100 to 1,000 cPs), ranging from 50 pg/mL to 1,000 pg/mL, and subsequently recorded the corresponding *A*_450nm_ values. The observed recovery rates were satisfactory, ranging from 99.5% to 105.1% for anti-SARS-CoV-2 NP IgA antibody detection, 97.1% to 102.4% for RBD detection, and 96.1% to 105.6% for SARS-CoV-2 Delta S1P detection, with all RSD values below 10% (Table S1). These results indicate that the MNP-based SenBox has sufficient accuracy for detecting protein biomarkers in saliva without interference.

**Table 1:** Determination of Spiked Anti-SARS-CoV-2 NP IgA Antibody, RBD, and SARS-CoV-2 Delta S1P in Human Saliva Was Conducted Using the MNP-based SenBox Combined with a Portable ColorReader to Record the b* Values and Calculate the Recovery Rates (*n* = 9)

spiked lgA					spiked RBD					spiked delta S1P				
sample	spiked conc. (pg/mL)	found (pg/mL)	recovery (%)	RSD (%)	sample	spiked conc. (pg/mL)	found (pg/mL)	recovery (%)	RSD (%)	sample	spiked conc. (pg/mL)	found (pg/mL)	recovery (%)	RSD (%)
human saliva	50	52.24	104.49	4.56	human saliva	50	51.53	103.06	4.03	human saliva	50	50.58	101.16	4.15
	250	246.17	98.47	4.44		250	247.71	99.09	3.11		250	251.37	100.55	3.61
	1,000	992.57	99.26	3.01		1,000	1,032.38	103.24	4.46		1,000	1,047.94	104.79	3.72

**Figure 5:**
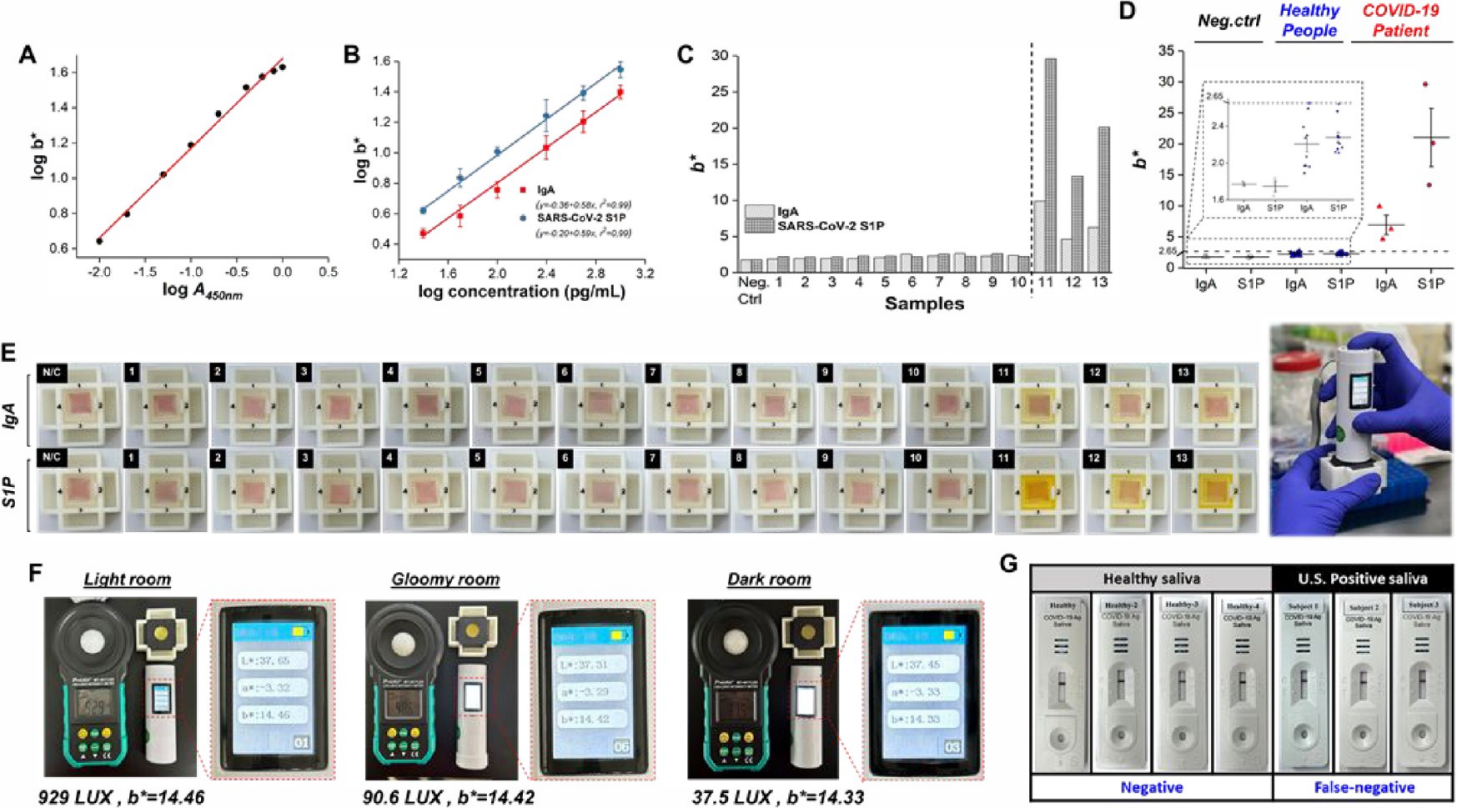
(A) The correlation between the colorimetric b* value and *A*_450nm_ value demonstrates the precision of the portable ColorReader (*n* = 3). The correlation, with an r-squared value of 0.994, indicates a strong predictive relationship between the two measurement methods. (B) Linear calibration curves were constructed between the b* values and the concentrations of SARS-CoV-2 S1P (25 pg/mL to 1,000 pg/mL) or anti-SARS-CoV-2 NP IgA antibody (25 pg/mL to 1,000 pg/mL), recorded by a portable ColorReader (*n* = 3). (C) The level of SARS-CoV-2 S1P and anti-SARS-CoV-2 NP IgA antibody in viscous saliva samples from 10 healthy individuals and 3 COVID-19 patients detected by MNP-based SenBox combined with a portable ColorReader for COVID-19 detection. (D) The distribution of b* values across negative control groups and real samples postanalysis enables the determination of a b* cutoff value through panel testing and analysis to immediately identify infectious diseases. This study’s cutoff value of b* = 2.65 was statistically derived from 3 negative controls and 13 real samples, facilitating rapid detection. (E) Detailed images illustrate the trends in colorimetric differentiation of MNP-based SenBox for SARS-CoV-2 S1P and anti-SARS-CoV-2 NP IgA antibody. The visual assessment revealed that solutions from negative saliva tests remained clear, while positive samples displayed a distinct yellow color, underscoring the efficacy of the MNP-based SenBox MHCT platform for rapid infection risk evaluation through observable color change and +b* value. (F) The demonstration of stable b* readings from the portable ColorReader across various lighting conditions (different illuminance) validates its suitability for semiquantitative field analysis. (G) Comparing the sensitivity and accuracy of the developed MNP-based SenBox with commercially available lateral flow test strips in detecting saliva samples obtained from healthy individuals (S1–S4) and COVID-19 patients (S11–S13). ^#^ IgA: anti-SARS-CoV-2 NP IgA antibody.

Calculating biomarker concentrations in a liquid biopsy by analyzing the *A*_450nm_ value of the postreaction solution using a spectrophotometer is a well-established and widely accepted method. The standard curves generated for anti-SARS-CoV-2 NP IgA antibody, RBD, SARS-CoV-2 Delta S1P, and SARS-CoV-2 Omicron S1P using the MNP-based SenBox with a microplate spectrophotometer have validated a positive correlation between the *A*_450nm_ values and the concentrations of these biomarkers (Figure 4C). To demonstrate that the portable ColorReader (Color Reader CR3; detailed specifications are presented in Table S2) can substitute for a microplate spectrophotometer in quantification for future mobile healthcare, we analyzed the correlation between the colorimetric b* values (where a higher b* value indicates more pronounced yellow coloration) obtained using the portable ColorReader and the *A*_450nm_ values obtained using the microplate spectrophotometer. The results demonstrated a highly positive correlation between b* values and *A*_450nm_ values (r^2^ = 0.994), thereby validating the reliability of the portable ColorReader employed for quantifying the concentrations of anti-SARS-CoV-2 NP IgA antibody, RBD, SARS-CoV-2 Delta S1P, and SARS-CoV-2 Omicron S1P in the viscous saliva samples (Figure 5A). Hence, we assessed the linear detection range of MNP-based SenBox for anti-SARS-CoV-2 NP IgA antibody and SARS-CoV-2 S1P by portable ColorReader. The standard curve demonstrated linearity within the 25 pg/mL to 1,000 pg/mL range (r^2^ = 0.996) for anti-SARS-CoV-2 NP IgA antibody detection and 25 pg/mL to 1,000 pg/mL range (r^2^ = 0.997) for SARS-CoV-2 S1P detection (Figure 5B).

Finally, a total of 10 saliva samples were collected from healthy individuals and 3 collected from the patients in the initial phases of SARS-CoV-2 infection to evaluate the efficacy of our MNP-based SenBox. Initially, the MNP-based SenBox was employed to detect anti-SARS-CoV-2 NP IgA antibodies and SARS-CoV-2 S1P in saliva samples from both healthy individuals and SARS-CoV-2-infected patients. A portable ColorReader was used to analyze the b* value of the colored solution, enabling the quantification of protein biomarker concentrations (Figure 5C and 5E). Salivary samples obtained from healthy individuals revealed no statistically significant difference in b* values pertaining to anti-SARS-CoV-2 NP IgA antibody and SARS-CoV-2 S1P when compared to the negative control group. Conversely, salivary samples obtained from individuals infected with SARS-CoV-2 exhibited a noticeable disparity in b* values associated with anti-SARS-CoV-2 NP IgA antibody and SARS-CoV-2 S1P in comparison to the negative control group (Figure 5C and 5E). Furthermore, statistical analysis was conducted by measuring the b* values of the MNP-based SenBox for 3 negative control samples (PBS) and 13 clinical saliva samples, utilizing a portable ColorReader (Figure 5D). Scatter interval plots illustrated that all b* values associated with the detection of anti-SARS-CoV-2 NP IgA antibody and SARS-CoV-2 S1P, attributed to both the negative control group (depicted in white) and healthy individuals (depicted in blue), remained below the threshold of 2.65. Conversely, all b* values linked to anti-SARS-CoV-2 NP IgA antibody and SARS-CoV-2 S1P detection from patients confirmed to be infected with SARS-CoV-2 via PCR surpassed 2.65 (depicted in red). These observations imply that the portable ColorReader may potentially replace traditional spectrophotometers for accelerated analysis of color development by the MNP-based SenBox, enabling the determination of infection status or even semiquantitative assessment of infection progression based on concentration determination.

While the exploration of utilizing smartphones for colorimetric detection and subsequent analysis of color developments is an active area of research, numerous unresolved challenges persist. For instance, utilizing a smartphone camera to capture the color-developed image and assess its grayscale value—derived from RGB values, as depicted in Figure S17—is prone to fluctuations induced by external lighting conditions, diverse smartphone manufacturers, varying focal lengths and angles of capture, thereby resulting in an inconsistent positive linear relationship between grayscale values and target concentrations compared to spectrophotometric analysis. Consequently, semiquantitative analysis may only be feasible under supplementary standardized photographic conditions. Conversely, we utilize the MNP-based SenBox to detect SARS-CoV-2 S1P in saliva samples from the SARS-CoV-2-infected patient and analyze the developed color after detection using a ColorReader under various environmental light conditions (LUX = 929, 90.6, and 37.5). The results showed that the obtained b* values (14.46 for 929 LUX; 14.42 for 90.6 LUX; 14.33 for 37.5 LUX) are not affected by the brightness of the detection environment (Figure 5F), indicating that the portable ColorReader is more suitable than smartphones for analyzing color signals based on colorimetric detection methods in environments with varying brightness. Furthermore, we used FDA-approved commercial saliva lateral flow rapid test strips to analyze clinical samples confirmed as COVID-19 positive by RT-PCR. The results showed that the strips were inadequate for detecting early stage infections, leading to false-negative outcomes, as demonstrated in Figure 5G. In contrast, the MNP-based SenBox developed in this study accurately detected all positive samples within 30 min. These findings suggest that the MNP-based SenBox offers superior sensitivity and specificity compared to traditional commercial lateral flow immunoassay rapid test strips. To further validate the detection accuracy of the MNP-based SenBox, we compared the concentrations of anti-SARS-CoV-2 NP IgA antibodies and SARS-CoV-2 S1P detected in clinical positive saliva samples using the MNP-based SenBox with those measured by ELISA (Table S3). The results showed no significant difference between the two methods, indicating that the detection accuracy of the MNP-based SenBox is sufficient.

### Detection and Quantification of Cytokines in a Rat Model of CFA-Induced Inflammation

**Figure 6:**
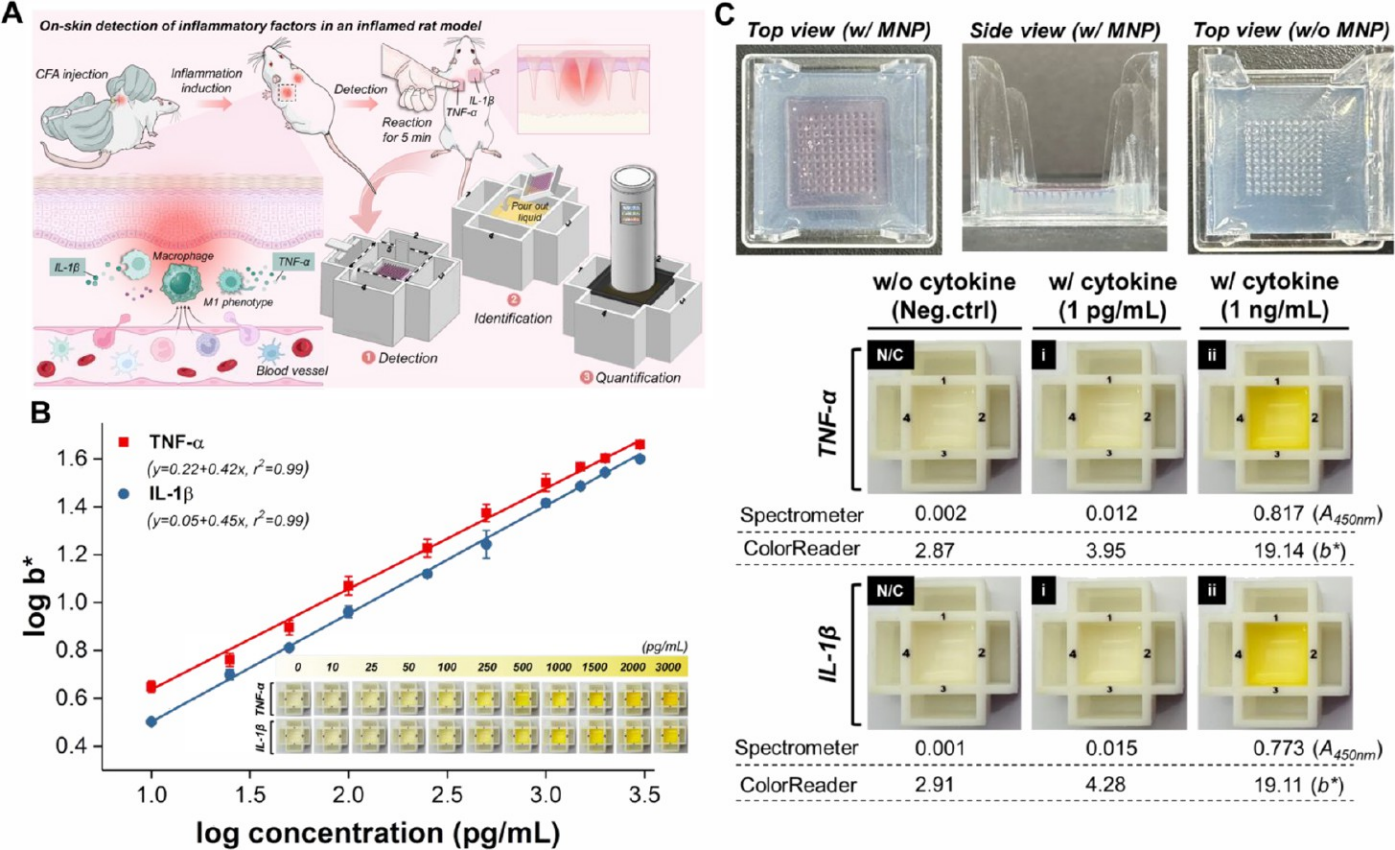
(A) Schematic showing the working principle of inflammation level induced by CFA administration, cytokine (TNF-α and IL-1β) generation, and application of the MNP-based SenBox for longitudinal detection. (B) Linear calibration curves were constructed between the b* values and the concentrations of TNF-α (10 pg/mL to 3,000 pg/mL) or IL-1β (10 pg/mL to 3,000 pg/mL), recorded by a portable ColorReader (*n* = 3). The inset displays the concentration-dependent color changes of the detection results using MNP-based SenBox. (C) Representative digital images of 1% agarose gel before and after insertion by MNP. The detailed images illustrate the trends in colorimetric differentiation of the MNP-based SenBox for TNF-α and IL-1β at different concentrations (1 pg/mL and 1 ng/mL).

Subsequently, to demonstrate the broad applicability of our MNP-based SenBox, we aimed to show that it can be used not only for noninvasive on-needle detection of protein biomarkers in liquid biopsy samples but also for the direct detection of protein biomarkers within the epidermal layer of skin. Frequent and timely measurement of protein biomarkers is crucial for disease monitoring and diagnostics in both biomedical research and clinical applications. However, conventional longitudinal measurements necessitate frequent blood draws over a short period, which may lead to iatrogenic anemia and increase patient morbidity. Additionally, repeated blood draws from small experimental animals are often impractical and can result in their death. To this end, we aimed to detect cytokines (TNF-α and IL-1β) within the epidermal layer of skin in a rat model of Complete Freund’s Adjuvant (CFA)-induced local inflammation. Using the MNP-based SenBox, we can perform frequent, sensitive, and accurate longitudinal measurements of cytokines (TNF-α and IL-1β) in the same rat, enabling us to monitor dynamic changes in cytokine levels and the immune system over time without requiring blood sampling (Figure 6A). We measured the concentrations of rat TNF-α and IL-1β within epidermal layer of skin using TNF-α-capture antibody (Ab_TNF-α_, A0277, ABclonal Inc., USA) or IL-1β-capture antibody (Ab_IL-1β_, A16288, ABclonal Inc., USA) functionalized MNP (Ab_TNF-α_@MNP or Ab_IL-1β_@MNP) and Ab_TNF-α_ or Ab_IL-1β_ functionalized HRP@ZIF-8 (Ab_TNF-α_-HRP@ZIF-8 or Ab_IL-1β_-HRP@ZIF-8). To achieve quantitative analysis, we assessed the linear detection range of MNP-based SenBox for TNF-α (RP01322, ABclonal Inc., USA) and IL-1β (RP01788, ABclonal Inc., USA) by portable ColorReader. The standard curve demonstrated linearity in the range of 10 pg/mL to 3,000 pg/mL for both TNF-α (r^2^ = 0.99) with a LOD of 4.5 pg/mL and IL-1β (r^2^ = 0.99) with a LOD of 9.4 pg/mL (Figure 6B). Additionally, we summarized the linear range and limit of detection (LOD) of our MNP-based SenBox in comparison with previously published studies and established gold-standard methods for TNF-α and IL-1β detection (Table S4) to highlight the relative advantages and limitations of our MNP-based SenBox in the broader context of biosensing technologies for inflammatory markers.

**Figure 7:**
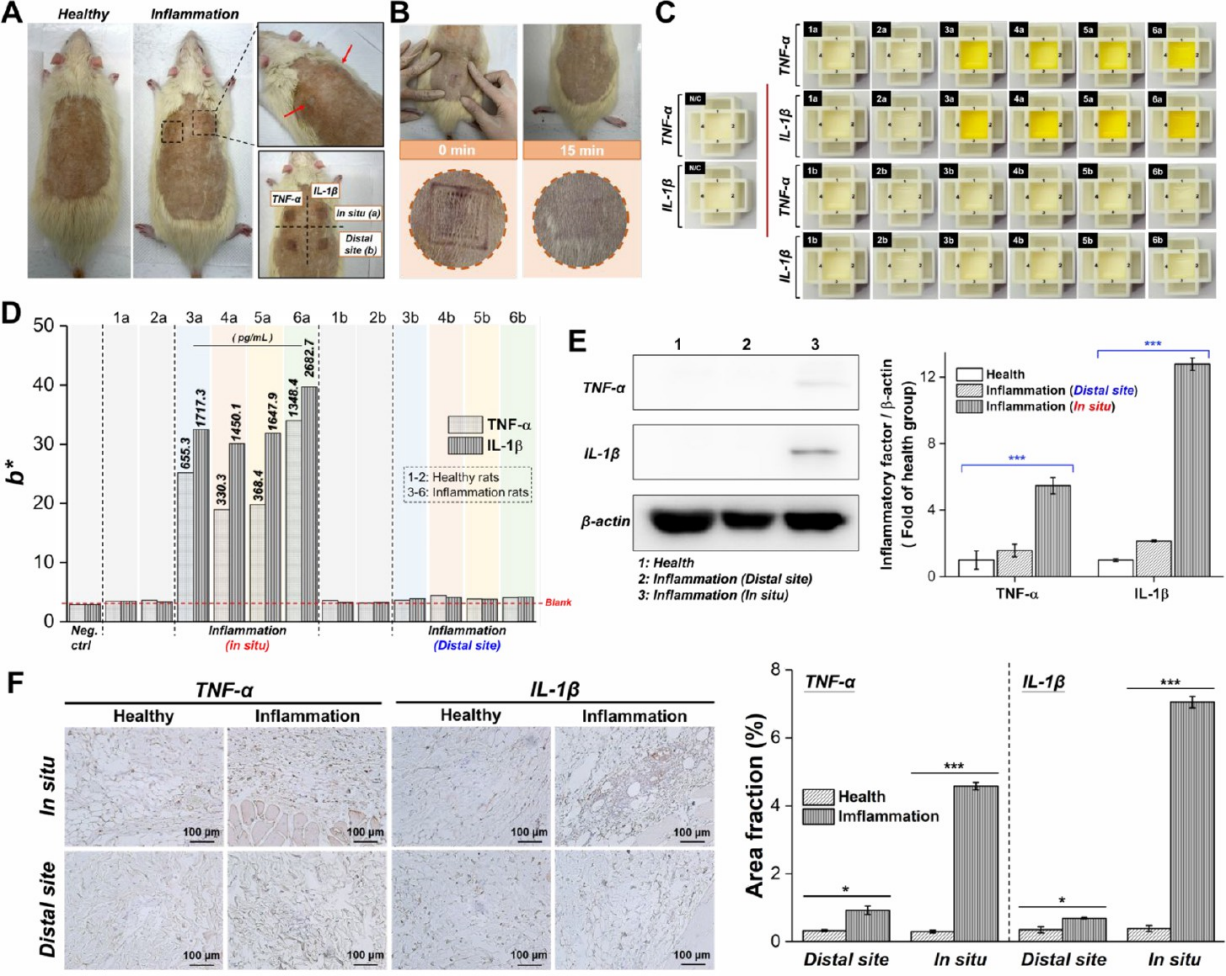
(A) Representative photographs of rats before and after inflammation induction by CFA administration, with the lower right photograph showing the application of Ab_TNF-α_@MNP and Ab_IL-1β_@MNP to various sites [*in situ (a)* and *distal site (b)* of inflammation] on the dorsal skin of rats with skin inflammation. (B) Rat skin puncture marks were observed 15 min following the removal of MNP. (C) Detailed images illustrate the detection results in color development of MNP-based SenBox for TNF-α and IL-1β within the epidermal layer of skin. N/C: Control group (MNP only); 1a-2a: *In situ* of healthy rat group; 1b-2b: *Distal site* of healthy rat group; 3a-6a: *In situ* of inflammatory rat group; 3b-6b: *Distal site* of inflammatory rat group. (D) The levels of TNF-α and IL-1β within the epidermal layer of two healthy rats and four rats with skin inflammation were detected using an MNP-based SenBox combined with a portable ColorReader for longitudinal inflammation monitoring. (E) Western blot (left) and quantitative analysis (right) of TNF-α and IL-1β levels for healthy rats and inflammatory rats (*n* = 4). ***indicates a significant difference (Student’s *t*-test, ***p ≤ 0.01). (F) IHC staining (left) and quantitative analysis (right) of TNF-α and IL-1β levels for healthy rats and inflammatory rats (*n* = 4). *indicates a significant difference (Student’s *t*-test, *p ≤ 0.05) and ***indicates a significant difference (Student’s *t*-test, ***p ≤ 0.01).

Before initiating animal studies, we initially conducted *in vitro* experiments to ascertain the practicality of using an MNP-based SenBox for the rapid *in vivo* capture and on-needle analysis of proteins within the epidermal layer of skin. The functionalized MNPs were applied to cytokine-spiked agarose gel and allowed to remain undisturbed for 5 min. After removing the MNPs from the agarose gel, on-needle analysis was performed to determine the concentrations of TNF-α and IL-1β using the SenBox. The results demonstrated that the Ab_TNF-α_@MNP or Ab_IL-1β_@MNP was capable of penetrating the agarose gel to capture the spiked TNF-α or IL-1β, and its b* value augmented with increasing spiked TNF-α and IL-1β concentrations (Figure 6C). Finally, Sprague–Dawley rats were injected dermally with CFA (containing 1 mg/mL of mycobacterial components) to induce skin inflammation. The Ab_TNF-α_@MNPs and Ab_IL-1β_@MNPs were administered to various sites (*in situ* inflammation site and distal site from inflammation site) on the dorsal skin of CFA-induced inflamed rats and left undisturbed for 5 min to capture the TNF-α and IL-1β within epidermal layer of skin followed by on-needle analysis using SenBox (Figure 7A). After the removal of the MNP, microindents on the skin were clearly visible due to the application of the MNP. However, these indents quickly diminished, with the skin returning to its normal state within 15 min, without any notable erythema, edema, or other adverse effects (Figure 7B). The MNPs retrieved from the dorsal skin of healthy rats, as well as from the dorsal skin at a site distal to the inflammatory area in CFA-induced inflamed rats, were subsequently probed *ex vivo* with Ab_TNF-α_-HRP@ZIF-8 or Ab_IL-1β_-HRP@ZIF-8 to quantify the concentrations of TNF-α and IL-1β using the SenBox. No statistically significant differences in b* values were observed compared to the negative control. In contrast, significant yellow coloration and markedly higher b* values were observed after on-needle analysis of the MNPs retrieved from dorsal skin at the inflammatory site in CFA-induced inflamed rats. On the basis of the standard curve (obtained using Ab_TNF-α_@MNP and Ab_IL-1β_@MNP exposed to known concentrations of TNF-α and IL-1β), the concentrations of TNF-α and IL-1β in the epidermal tissues at the inflammatory site in rats were determined to be in the range of 330.3–1348.4 pg/mL and 1450.1–2682.7 pg/mL, respectively (Figure 7C and 7D). The results were further validated using a commercial spectrophotometer, which was employed to measure the generated signal and determine the concentrations of TNF-α and IL-1β in the epidermal tissues, as presented in Table S5. The minimally invasive MNP-based SenBox represents a transformative approach to perform frequent, sensitive and accurate measurements of protein biomarkers within epidermal layer of skin. Additionally, the concentration of IL-1β is significantly higher than that of TNF-α in the epidermal tissues of rats with CFA-induced inflammation, likely because we conducted direct epidermal biosensing of TNF-α and IL-1β 1 day after inducing the inflammatory response. The release of TNF-α occurs almost immediately after the onset of inflammation, usually peaking within hours, followed by the release of large amounts of IL-1β, which peaks within days of the inflammatory response.^38^ To confirm the successful induction of inflammation and to verify the accuracy of the inflammatory cytokine concentration trends detected by the MNP-based SenBox, we examined the expression of TNF-α and IL-1β in various skin tissue regions of CFA-induced inflamed rats using Western blot analysis (Figure 7E) and immunohistochemistry (IHC; Figure 7F). The results indicated that, compared to healthy rats, the CFA-induced inflammation area significantly increased TNF-α expression by 5.47 ± 0.49-fold and IL-1β expression by 12.78 ± 0.38-fold. Mild inflammation was also observed in the skin distant from the site of inflammation induction, with lower expression levels of TNF-α and IL-1β. It was also found that the concentration of IL-1β at the inflammation site was significantly higher than that of TNF-α. These results are consistent with the trends detected by the MNP-based SenBox for rapid detection of TNF-α and IL-1β concentrations in epidermal tissues, confirming that the MNP-based SenBox is not only capable of providing rapid, real-time, and accurate detection of protein biomarkers in epidermal tissues but also minimizes patient discomfort and the risk of infection, making it highly suitable for mobile healthcare.

Compared to similar published works, our MNP-based SenBox offers several distinct advantages regarding workflow efficiency and ease of use. Traditional microneedle systems often serve as sampling tools, necessitating off-device analysis using sophisticated laboratory equipment, which can be time-consuming and resource-intensive.^17^ In contrast, our system integrates sample collection with real-time on-needle analysis, eliminating the need for extensive off-device processing and enabling rapid results in under 30 min. This streamlined workflow makes our approach particularly suitable for point-of-care testing (POCT) applications, significantly reducing the overall assay time.^39^ Additionally, our device requires minimal training due to the portable ColorReader, which provides a user-friendly interface for biomarker quantification. This contrasts with systems that need specialized personnel or equipment, making our method more accessible in resource-limited settings. However, while our system is highly effective for targeted biomarker detection, it is not yet optimized for continuous monitoring or multiplexed analysis, which are areas where other microneedle-based biosensors may have advantages.^40^

Nonetheless, the versatility of our device in handling both viscous liquid biopsies and transdermal biosensing sets it apart from other systems that focus on a single application type. This flexibility, combined with the rapid and minimally invasive nature of the MNP-based SenBox, underscores its potential for broad clinical and mobile healthcare applications. Overall, this concept can be applied across various fields to analyze target protein biomarkers and understand disease progression in cancers, chronic diseases, and infectious diseases. These results suggest that the MNP-based SenBox is adequate for evaluating protein biomarker levels, enabling comprehensive proteomic and metabolomic analysis through *ex vivo* and *in vivo* biosensing.

## Conclusions

In conclusion, we have developed a comprehensive biosensing technology based on the MNP-based SenBox, integrated with biomineralization-enhanced colorimetric analysis, capable of detecting protein biomarkers across various liquid biopsy types through *ex vivo* or direct *in vivo* biosensing. Combined with a portable ColorReader, this system enables regular quantification of protein content variations in liquid biopsies, facilitating longitudinal monitoring. Using saliva samples from patients in the early stages of SARS-CoV-2 infection and a skin inflammation rat model, we demonstrate that the MNP-based SenBox allows for straightforward and timely detection of relevant protein biomarkers in liquid biopsies. Additionally, it supports at-home sampling and centralized biomarker level detection through self-administration of the MNP, enabling timely therapy monitoring. The MNP-based SenBox, optimized with HRP@ZIF-8, achieves a broad detection range from 5 pg/mL to 1,000 pg/mL for SARS-CoV-2 S1P, and from 10 pg/mL to 1,000 pg/mL for anti-SARS-CoV-2 NP IgA antibody, TNF-α, and IL-1β, all within 30 min and without the need for professional training. While the MNP-based SenBox offers a portable, minimally invasive solution for protein biomarker detection, it has not yet been optimized for continuous monitoring or multiplexed biomarker detection using a single MNP. Future improvements could focus on enhancing its capabilities for continuous monitoring, enabling multiplexed detection, expanding the range of detectable analytes, and integrating wireless communication for real-time data transmission, thereby facilitating broader clinical applicability and point-of-care diagnostics. Nevertheless, the MNP-based SenBox MHCT has demonstrated significant potential for applications across a wide range of diseases, particularly in mobile healthcare and resource-limited settings, enabling rapid disease diagnosis and efficient therapeutic intervention in a patient-friendly manner.

## Methods

### Preparation of AuNPs-Coated Layer-by-Layer 3D Microneedles

Ten g of PLLA was dissolved in 100 mL of dichloromethane (DCM). The solution was then agitated on a rotary shaker at 500 rpm and kept in the dark at room temperature for 1 h. Subsequently, 0.25 mL of (3-Aminopropyl)triethoxysilane (APTES) was slowly added to the solution, and the mixture was allowed to react in the dark for 16 h. The resulting mixture was then spread evenly on a glass slide to dry, forming a thin film stored in a sealed condition.

Cut the prepared APTES-PLLA film into approximately 1 × 1 cm squares and place them flat onto a PDMS mold. Utilize the hot-press molding technique to heat the film to 190 °C, allowing it to melt and flow into each cavity of the PDMS mold, then cool it to solidify. Finally, the APTES-silanized PLLA MNP with amino (−NH_2_) functional groups from the PDMS mold was demolded.

LBL_PSS/PAH_-PLLA MNP was prepared using negatively charged poly(sodium 4-styrenesulfonate) (PSS) and positively charged poly(allylamine hydrochloride) (PAH) through the LBL assembly technology based on electrostatic interaction. PSS/PAH base layers were deposited through alternate immersion into PSS (2 mg/mL in 0.1 M NaCl) and PAH (2 mg/mL in 0.1 M NaCl) for 30 min, separated by two 1 min PBS rinses to form LBL_PSS/PAH_-PLLA MNP. Subsequently, the LBL_PSS/PAH_-PLLA MNP was dipped in a solution of citrate-capped AuNPs (optical density at 530 nm was 0.5) and incubated for 30 min at room temperature to allow the AuNPs to self-assemble on the LBL_PSS/PAH_-PLLA MNP surface to form AuNPs@MNP. After the coating process, the AuNPs@MNP underwent three rinses with PBS to remove any unattached AuNPs effectively. Finally, to preserve their integrity and ensure their readiness for future experimental applications, the AuNPs@MNP was stored at a temperature of 4 °C until further use. The surface morphology of the fabricated AuNPs@MNP was confirmed by SEM (SU8220, Hitachi).

### Preparation of Biorecognition Element-Modified MNP

To prepare NP@MNP (for anti-SARS-CoV-2 NP IgA antibody detection) and Ab_RBD_@MNP (for SARS-CoV-2 S1P detection), the recombinant SARS-CoV-2 NP (LDG006PVM, Leadgene Biomedical, Inc., Taiwan) and rabbit polyclonal anti-RBD antibody (Ab_RBD_; A20135, ABclonal Inc., USA) must first be modified with thiol groups. Briefly, SARS-CoV-2 NP and Ab_RBD_ were reacted with 100 mM 2-iminothiolane hydrochloride (Traut’s reagent) in PBS (pH 7.4; containing 2 mM EDTA) at room temperature for 1 h. Then, excess Traut’s reagent was removed by a dextran desalting column, and the concentrations of the eluted thiol-modified SARS-CoV-2 NP and Ab_RBD_ (SH-SARS-CoV-2 NP and SH-Ab_RBD_) were quantified using a BCA Protein Assay Kit. The collected SH-SARS-CoV-2 NP and SH-Ab_RBD_ were stored at −20 °C until further use. Afterward, the prepared AuNPs@MNP were then reacted with SH-SARS-CoV-2 NP (1 mL of 0.25 ng/μL) and SH-Ab_RBD_ (1 mL of 0.25 ng/μL) at room temperature for 1 h to conjugate with AuNPs attached to the AuNPs@MNP surface to form NP@MNP and Ab_RBD_@MNP. After washing with PBS, the residual SH-SARS-CoV-2 NP and SH-Ab_RBD_ in the supernatant were then quantified by an ELISA kit to determine the concentration of SH-SARS-CoV-2 NP and SH-Ab_RBD_ immobilized on the AuNPs@MNP. The NP@MNP and Ab_RBD_@MNP were blocked using 1% bovine serum albumin (BSA) in PBS for another 1 h at room temperature and then washed three times with TBST to remove unreacted BSA. The obtained NP@MNP and Ab_RBD_@MNP were stored at a temperature of 4 °C until further use.

### Preparation of HRP-Incorporated Zeolitic Imidazolate Frameworks (HRP@ZIF-8)

The synthesis of zeolitic imidazolate frameworks (ZIF-8), especially when modified with antibodies and encapsulated with Horseradish Peroxidase (HRP), is an intricate process that demands precision. To initiate the synthesis of ZIF-8 and HRP@ZIF-8, 25 mg of HRP and 5 mg of polyvinylpyrrolidone (PVP with an average Mw of 10,000 Da) were added to a 40 mL aqueous solution of 2-Methylimidazole (2-MIM) at a concentration of 1.22 M. The mixture was then stirred for 10 min at room temperature. For the preparation of pure ZIF-8, adding HRP is unnecessary. The subsequent step involves slowly adding 4 mL of ZnCl_2_ (100 mg/mL) to the stirred mixture. A stirring speed of 500 rpm is maintained, and the reaction can proceed for 30 min before leaving it undisturbed overnight. The product is centrifuged at 12,000 rpm for 10 min after the reaction. This step is pivotal for removing any unreacted species from the mixture. The products obtained at this juncture are termed as either ZIF-8 or HRP@ZIF-8. For storage purposes, these products are refrigerated at 4 °C. When these products are required for further utilization, they are redissolved in an aqueous solution of 0.01% poly(vinyl alcohol) (PVA with an average Mw of 6,000 Da).

To prepare antibody-modified HRP@ZIF-8 (sAb_IgA_-HRP@ZIF-8 and Ab_RBD_-HRP@ZIF-8), the synthesized HRP@ZIF-8 was first diluted using DI-H_2_O to achieve the desired concentration (0.677 mg/mL). According to thermogravimetric analysis (TGA) results, HRP comprised approximately 17.3 wt %, resulting in a final HRP concentration of 117 μg/mL. Thiolated antibodies (SH-sAb_IgA_ and SH-Ab_RBD_) were introduced into this diluted solution, ensuring a final 100 ng/mL concentration. The mixture (1 mL) was allowed to react at room temperature in the dark for 2 h. A centrifuge was then used to remove unreacted components in the supernatant. The obtained pellet was subsequently redispersed in a 0.5 wt % BSA blocking buffer and allowed to react for 1 h. This step is essential as it curbs the potential for nonspecific adsorption. After the reaction, another centrifugation was conducted to discard the supernatant, followed by washing the pellet with TBST solution. Ultimately, the pellet was redissolved in PBS (1 mL), transforming it into a signal probe for detection assays.

The X-ray absorption fine structure (XAFS) of the Zn K-edge of samples was carried out at the SPring-8 (Japan) 12B2 Taiwan beamline of the National Synchrotron Radiation Research Center (NSRRC) operated at 8.0 GeV with a constant current of approximately 100 mA.

### Schematic Functionality of the Rapid Biomarker POCT in Viscous Saliva Samples Using MNP-Based SenBox for COVID-19 Testing

All samples used in this study were collected with the approval of the Chang Gung Medical Foundation Human Experiment Ethics Committee for clinical trials and research. The IRB case number for SARS-CoV-2 detection is 202000986B0. Additionally, the storage and transport of serum specimens adhered to the guidelines set by the Centers for Disease Control and Prevention in Taiwan (Taiwan CDC). The intricate design and functionality of the rapid biomarker POCT using the MNP-based SenBox are depicted in Figure 1. The MNP-based SenBox features a tray equipped with a handle that securely nests the MNP. This MNP is preserved between uses by a protective sealing film within the central slot of the SenBox, ensuring sterility and reagent integrity.

The assay is initiated by depositing a saliva sample onto the tray-mounted MNP for 15 min, after which it is sequentially engaged with four distinct solution-filled slots by tearing their sealing films in a specified order. Each slot has a dedicated function that facilitates a multistep process for biomarker detection:

1. First wash buffer slot (slot 1): The tray is placed in the wash buffer slot to remove nonspecifically bound impurities and residual saliva from the MNP, ensuring a clean surface for specific immunological binding.
2. Signal probe solution slot (slot 2): The tray is then immersed in the signal probe solution (1 mL) for 15 min, allowing the formation of a sandwich immunocomplex on the MNP surface. The HRP@ZIF-8-based signal probes (sAb_IgA_-HRP@ZIF-8 or Ab_RBD_-HRP@ZIF-8) bind to the MNP in the presence of target proteins.
3. Second and third wash buffer slots (slots 3 and 4): These slots are used for subsequent immersion to cleanse the MNP by removing unbound HRP@ZIF-8-based signal probes (sAb_IgA_-HRP@ZIF-8 or Ab_RBD_-HRP@ZIF-8). This step is crucial for ensuring the specificity of the assay.
4. Monitoring slot (slot 5): Finally, the tray is returned to the central slot to add the H_2_O_2_/TMB substrate. After 3 min, the resulting colorimetric reaction is stopped by adding HCl, and the liquid in the tray is poured into slot 5. The test results can be assessed visually, with colorless indicating a negative result and yellow indicating a positive result. Alternatively, a portable ColorReader can be used to measure the b* value, allowing determination of the concentrations of anti-SARS-CoV-2 NP IgA antibody and anti-SARS-CoV-2 NP based on the measured b* value.

### Detection of TNF-α and IL-1β in an Inflammatory Rat Model

This study was conducted following a protocol approved by the Institutional Animal Research Committee at Kaohsiung Medical University (IACUC number: 113046). Male Sprague–Dawley rats weighing approximately 450 g were procured from BioLASCO in Taipei, Taiwan. Upon arriving at the Laboratory Animals at Kaohsiung Medical University, the rats were acclimated for at least 1 week before beginning the experiments. They were housed in a controlled environment with a 12-h light/dark cycle, a consistent temperature of 22.1 °C, and 55% relative humidity, with unrestricted access to standard feed and water throughout the study. To induce skin inflammation, Complete Freund’s Adjuvant (CFA) (InvivoGen; vac-cfa-10; San Diego; USA) containing 1 mg/mL of mycobacterial components was used. Before the injection, the animals were anesthetized with an intramuscular injection of Zoletil 50 (Virbac; Zoletil 50; Carros; France) at a dosage of 0.1 g/100 g body weight. The injections with CFA were administered dermally on shaved areas of the skin, which were thoroughly cleaned with povidone-iodine scrub and 70% alcohol before the procedure.

The Ab_TNF-α_@MNP and Ab_IL-1β_@MNP were inserted into the dorsal skin of healthy rats and inflamed rats by pressing against the backside of an MNP with a thumb using a force of approximately 1.5 N and then removed after 5 min of insertion. The removed MNP was then put on the tray and sequentially engaged with four distinct, solution-filled slots by tearing their sealing films in a specified order. The slots, each with a dedicated function, facilitate a multistep process for biomarker detection:

1. First wash buffer slot (slot 1): The tray is placed in the wash buffer slot to remove nonspecifically bound impurities and residual ISF from the MNP, ensuring a clean surface for specific immunological binding.
2. Signal probe solution slot (slot 2): The tray is then immersed in the signal probe solution (1 mL) for 15 min, allowing the formation of a sandwich immunocomplex on the MNP surface. The HRP@ZIF-8-based signal probes (Ab_TNF-α_-HRP@ZIF-8 or Ab_IL-1β_-HRP@ZIF-8) bind to the MNP in the presence of target proteins.
3. Second and third wash buffer slots (slots 3 and 4): These slots are used for subsequent immersion to cleanse the MNP by removing unbound HRP@ZIF-8-based signal probes (Ab_TNF-α_-HRP@ZIF-8 or Ab_IL-1β_-HRP@ZIF-8). This step is crucial for ensuring the specificity of the assay.
4. Monitoring slot (slot 5): Finally, the tray is returned to the central slot to add the H_2_O_2_/TMB substrate. After 3 min, the resulting colorimetric reaction is stopped by adding HCl, and the liquid in the tray is poured into slot 5. Subsequently, a portable ColorReader can be used to measure the b* value, allowing determination of the concentrations of TNF-α and IL-1β based on the measured b* value.

The entire assay, from initiation to result interpretation, is meticulously calibrated for accuracy, culminating in analysis via traditional spectrophotometry and a portable ColorReader. This novel POCT based on the MNP-based SenBox represents a significant advancement in the field of rapid diagnostics, promising a seamless transition from sample collection to result acquisition.

### Statistical Analysis

The data were expressed as the mean ± SD based on at least three independent experiments. Statistical analysis was performed using Student’s *t* test. Differences were considered statistically significant if *p ≤ 0.05 and ***p ≤ 0.01.

## Supporting Information Available

Experimental section, supplementary figures (S1–S17), and supplementary tables (S1–S5); (Figure S1) FTIR spectra of PLLA MNP and APTES-silanized PLLA MNP, (Figures S2 and S3) successful deposition of AuNPs on LBL_PSS/PAH_-PLLA MNP, (Figures S4–S6) successful conjugation of antibodies on the surface of AuNPs@MNP, (Figure S7) long-term stability and durability of NP@MNP and Ab_IL-1β_@MNP, (Figure S8) XRD pattern of ZIF-8 and HRP@ZIF-8, (Figure S9) TGA analysis of the ZIF-8 and HRP@ZIF-8, (Figure S10) BET surface area analysis of ZIF-8 and HRP@ZIF-8, (Figures S11 and S12) catalytic activity and stability analysis of HRP@ZIF-8, (Figure S13) comparative analysis of the stability of naked HRP and HRP@ZIF-8 under different environmental conditions, (Figure 14) stability evaluation of Ab_RBD_-HRP@ZIF-8 in PBS, (Figure S15) Michaelis–Menten kinetic analysis of HRP and HRP@ZIF-8, (Figure S16) evaluation of thiolated antibody conjugation onto HRP@ZIF-8 by ELISA assay, (Figure S17) comparative analysis of image grayscale value differences across various smartphone brands, (Table S1) effects of sample viscosity on the detection accuracy of the MNP-based SenBox, (Table S2) specifications of Color Reader CR3, (Table S3) comparative analysis of detection accuracy between MNP-based SenBox and ELISA, (Table S4) comparison of cytokine detection methods in this study with previously published works, and (Table S5) comparison of TNF-α and IL-1β concentrations quantified by a commercial spectrophotometer and a portable ColorReader (PDF)
(Video S1) Complete workflow of the MNP-based SenBox (MP4)
