# Supplementary material for: Lab-on-the-Needles: A Microneedle Patch-Based Mobile Unit for Highly Sensitive Ex Vivo and In Vivo Detection of Protein Biomarkers
Source: ACS Nano. 2025 Jan 7;19(3):3249–64. doi: 10.1021/acsnano.4c11238 (PMC11781025; doi:10.1021/acsnano.4c11238)
Supplement: Supplementary file 1 — nn4c11238_si_001.pdf [file nn4c11238_si_001.pdf]

Supporting Information

Lab-on-the-Needles: A Microneedle Patch-Based Mobile Unit for Highly Sensitive *Ex Vivo* and *In Vivo* Detection of Protein Biomarkers

Ying-Pei Hsu[1,#], Nan-Si Li[2,#], Hao-Han Pang[2], Yu-Chi Pan[3], Hung-Pei Tsai[4], Hsiao-Chien Chen[5,6], Ying-Tzu Chen[2,7], Chen-Hsun Weng[8], Shiao-Wei Kuo[1,9,*], Hung-Wei Yang[2,8,*]

[1]Department of Materials and Optoelectronic Science, National Sun Yat-sen University, Kaohsiung 80424, Taiwan

[2]Department of Biomedical Engineering, National Cheng Kung University, Tainan 70101, Taiwan

[3]Graduate Institute of Medicine, College of Medicine, Kaohsiung Medical University, Kaohsiung 80708, Taiwan

[4]Division of Neurosurgery, Department of Surgery, Kaohsiung Medical University Hospital, Kaohsiung Medical University, Kaohsiung 80708, Taiwan

[5]Center for Reliability Science and Technologies, Chang Gung University, Taoyuan 33302, Taiwan

[6]Kidney Research Center, Department of Nephrology, Chang Gung Memorial Hospital, Linkou, Taoyuan 33305, Taiwan

[7]Department of Neurosurgery, Chang Gung Memorial Hospital, Linkou, Taoyuan 33305, Taiwan

[8]Medical Device Innovation Center, National Cheng Kung University, No. 1, University Rd., Tainan City 70101, Taiwan

[9]Department of Medicinal and Applied Chemistry, Kaohsiung Medical University, Kaohsiung, 80708, Taiwan

[#] Y. P. Hsu and N. S. Li contributed equally to this work.

Corresponding Author

*Tel: (+886)-7-5252000#4079; E-mail address: kuosw@faculty.nsysu.edu.tw

*Tel: (+886)-6-2757575#63421; E-mail address: howardyang@gs.ncku.edu.tw

Experimental Section

Fabrication of polydimethylsiloxane (PDMS) microneedle (MN) array mold

Polydimethylsiloxane (PDMS) is a composite material derived from a base and curing agent. The base and curing agents were mixed in a 10:1 mass ratio for mold fabrication. Specifically, 18 g of the base agent was combined with 1.8 g of the curing agent. This mixture was thoroughly stirred using a glass rod to ensure homogeneity. To eliminate any entrapped air bubbles that might compromise the mold quality, the mixture was placed in a vacuum oven for 30 min.

After degassing, the PDMS mixture was poured into a plastic container with an affixed microneedle master mold, ensuring complete coverage. Any tiny bubbles formed at this stage were removed using a bulb syringe. The container was then transferred to a heating plate set at 70°C, where the PDMS mixture was cured for 3 hr. Upon solidification, the PDMS was carefully demolded from the master mold in the plastic container. The resultant PDMS block was then trimmed to the desired size to produce the PDMS microneedle mold. The fabricated mold, after inversion, consisted of a 10 x 10 array of MNs. Each MN had a base width of approximately 893 μm and a total height of about 858 μm, with the needle portion measuring around 427 μm and the base section approximately 431 μm.

Before fabricating the MNP, the PDMS molds were placed in a beaker containing deionized water and cleaned using an ultrasonic cleaner for 30 min. The molds were then dried in an oven set at 80°C. Before material modification, the mold's surface was repeatedly dabbed with adhesive tape to ensure both the inner and outer surfaces of the PDMS molds were clean.

Analytical methodology and instrumentation for semi-quantitative biomarker concentration analysis

In this study, we employed the BioTek Epoch 2 microplate spectrophotometer as our standard instrument for measuring absorbance intensities at 450 nm (A_{450nm}) and conducting semi-quantitative analysis of anti-SARS-CoV-2 NP IgA antibody and SARS-CoV-2 S1P. The microplate spectrophotometer is a critical tool in biometric analysis due to its precision and reliability in generating measurement data. To further enhance our analysis capabilities, especially in POCT, we utilized the 3nh ColorReader, a handheld micro colorimeter known for its instantaneous and precise color measurement capabilities. The high-precision design of this portable device makes it an ideal choice for our study. Key features include non-contact automatic calibration to ensure long-term measurement accuracy and a multifunctional bright button for easy operation, independently or in conjunction with an app. Its integrated sphere dual optical path design augments the stability and accuracy of measurements. The device's compactness also allows for easy portability, fitting conveniently into a pocket. It includes a rechargeable lithium battery for prolonged use and supports Bluetooth 5.0 for stable data transmission. Compared to more expensive spectrometers, this device offers a high-quality industrial-grade real-time microcontroller unit at a lower cost (less than 200 USD), delivering test results within a second. The accompanying app provides various data display modes and access to an extensive color card database, rendering the ColorReader a versatile tool for professionals in the field of biosensing.

The detailed specifications of the ColorReader used in our study are presented in Table S2. This portable ColorReader supports up to 20 different color standards, suitable for the semi-quantitative analysis in our study, which involves color changes from transparent to yellow. These changes align with the CIE Lab color space proposed by the International Commission on Illumination (CIE). The CIE Lab color space, a mathematical model designed to compare color differences as perceived by the human eye, encompasses $L*$ representing luminance from -$L*$ (black) to +$L*$ (white), the $a*$ axis indicating hues from green (-*a*) to red (+$a*$), and the $b*$ axis from blue (-*b*) to yellow (+$b*$). This color

space is widely used in photography, printing, painting, and color management systems due to its ability to accurately reflect and compare color differences. Since the CIE Lab color space correlates with human color perception, it is particularly useful in color correction and management. In our study, we leveraged these instruments to analyze the color changes resulting from increased target concentrations. As the reactants shift from transparent to yellow, the color moves towards (+b^*), serving as the basis for our semi-quantitative point-of-care analysis methodology.

For TNF-α detection using the MNP-based SenBox, the linear regression is described by the equation $\log(y) = 0.42 \times \log(x) + 0.22$, where y represents the b^* value and x represents the concentration in pg/mL. Based on the measurements of the blank group ($b^* = 2.92$, SD = 0.055), the LOD was calculated using the formula LOD signal = $b^* + 3 \times$ SD (blank), resulting in a LOD of 4.5 pg/mL.

For IL-1β detection using the MNP-based SenBox, the linear regression is described by the equation $\log(y) = 0.45 \times \log(x) + 0.05$, where y represents the b^* value and x represents the concentration in pg/mL. Based on the measurements of the blank group ($b^* = 2.96$, SD = 0.042), the LOD was calculated using the formula LOD signal = $b^* + 3 \times$ SD (blank), resulting in a LOD of 9.4 pg/mL.

Western blot and immunohistochemical (IHC) analysis of skin tissues

We analyzed Western blot and IHC on skin tissues excised from healthy and inflamed rats to assess the inflammation levels in CFA-induced inflamed rats.

For Western blot analysis, the obtained rat skin tissues were lyophilized, ground into a powder, and then homogenized on ice using a Polytron blender in lysis buffer supplemented with a protease inhibitor cocktail. The homogenates were centrifuged at 2,000 rpm for 10 min at 4°C, and the supernatant was assayed for total protein concentration by BCA Protein Assay Kit and stored at −80 °C until used for TNF-α and IL-1β analysis using Western blotting. Proteins were electrophoresed using a 15% SDS-PAGE gel (approximately 50 μg per lane) and transferred to a polyvinylidene

fluoride (PVDF) membrane. After blocking with blocking solution (5% milk, 0.1% Tween-20 in TBS buffer, pH=7.4), the β-actin internal control was stained with β-actin monoclonal antibody [HRP-conjugated β-Actin rabbit mAb (AC028), ABclonal, 1:2000]. Target proteins (TNF-α and IL-1β) were stained with anti-TNF-α antibody [TNF-α rabbit pAb (A0277), ABclonal, 1:500] and anti-IL-1β antibody [IL-1β rabbit pAb (A16288), ABclonal, 1:500], respectively. A goat-anti-rabbit IgG (H+L)-HRP antibody was used as the secondary antibody for all proteins mentioned. Chemiluminescence signals were imaged using a ChemiDocTM XRS imaging system.

For IHC analysis, sections were immunostained with the antibodies at 4°C overnight, anti-TNF-α antibody [TNF-α rabbit pAb (A0277), ABclonal, 1:500] and anti-IL-1β antibody [IL-1β rabbit pAb (A16288), ABclonal, 1:500] against the TNF-α and IL-1β, respectively. The primary antibody was directed using a peroxidase-based kit (UltraVision Quanto, Epredia, USA) and visualized by 3,3′-diaminobenzidine (DAB, Epredia) substrate with enhancer. The sections were subsequently counterstained with hematoxylin. Digital images of the vessels were scanned using a light microscope (BX43, Olympus, Japan), and the density and intensity of the signal were determined by ImageJ software.

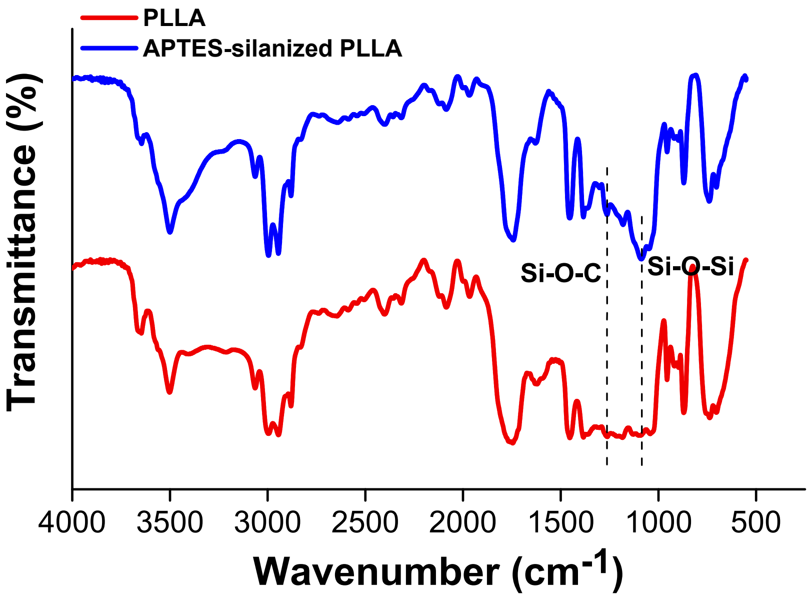

Figure S1. The FTIR spectra of PLLA MNP and APTES-silanized PLLA MNP.

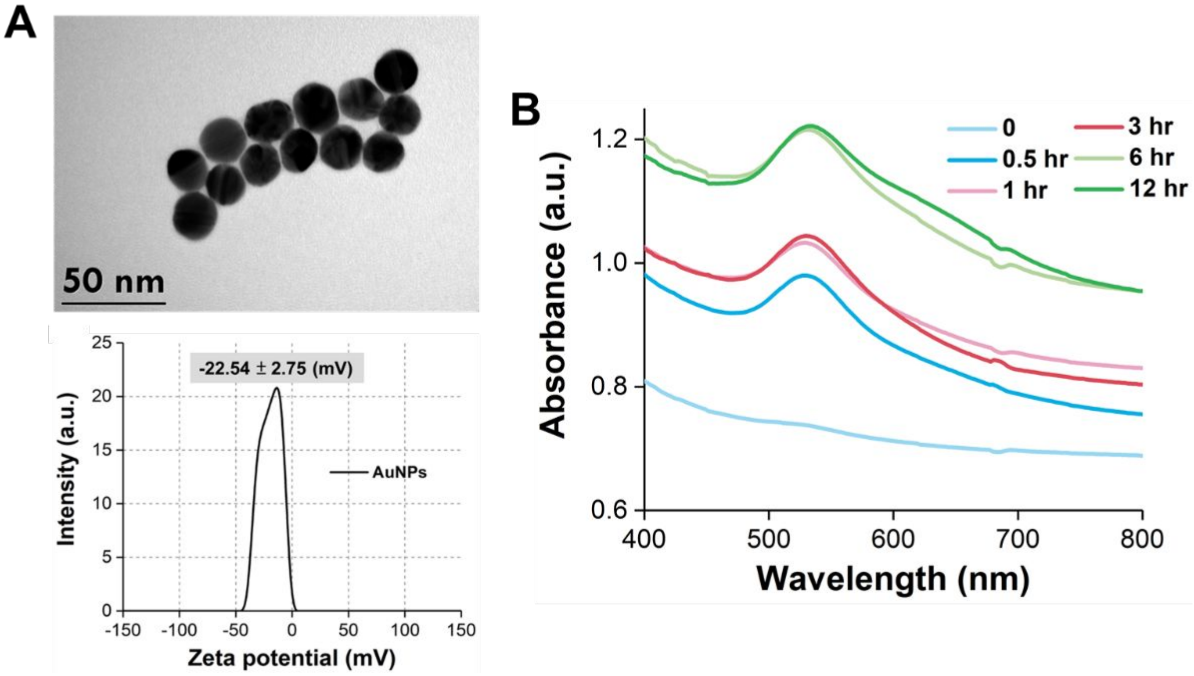

Figure S2. (A) TEM image and zeta potential analysis of AuNPs. (B) UV-Vis-NIR spectra of the AuNPs@MNP prepared by self-assembling AuNPs on $LBL_{PSS/PAH}$-PLLA MNP for 0.5-12 hr.

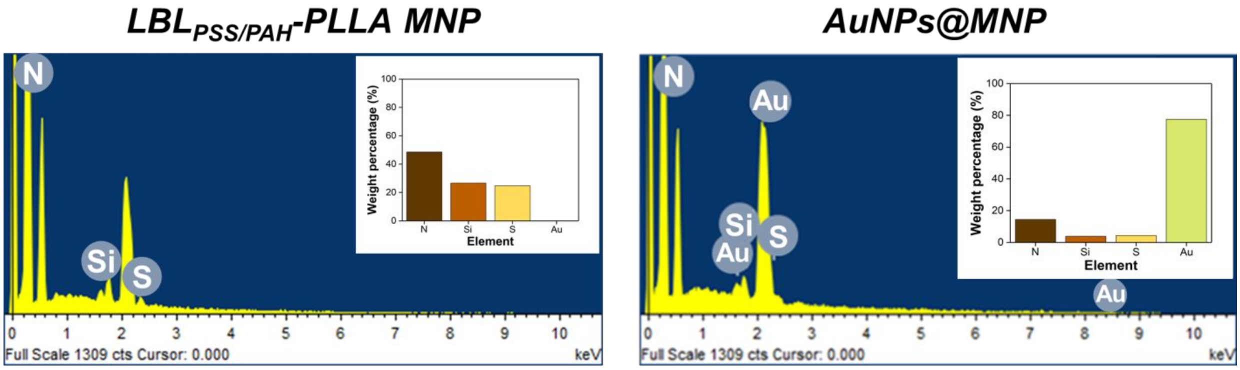

Figure S3. The EDS elemental analysis was conducted for LBL$_{PSS/PAH}$-PLLA MNP and AuNPs@MNP. Abundant Au, nitrogen, and sulfur elements were found, providing evidence of AuNPs on LBL$_{PSS/PAH}$-PLLA MNP.

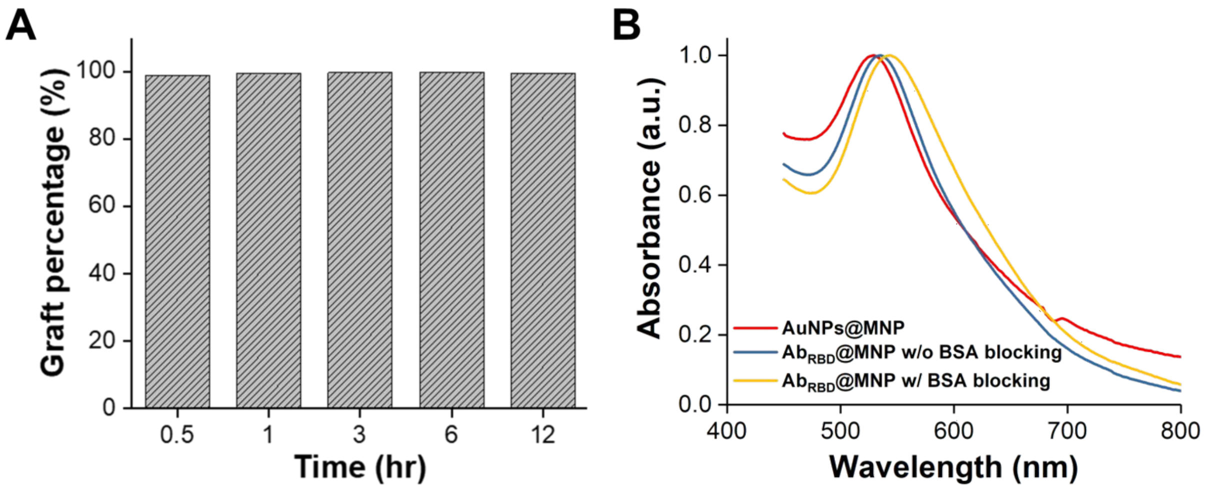

Figure S4. (A) A bar graph highlighting the conjugated rate of Ab$_{RBD}$ on the AuNPs@MNP after being coated with AuNPs for various durations (0.5, 1, 1.5, 3, 6, and 12 hr). (B) UV-Vis-NIR absorption spectra of AuNPs@MNP after Ab$_{RBD}$ conjugation and/or BCA blocking.

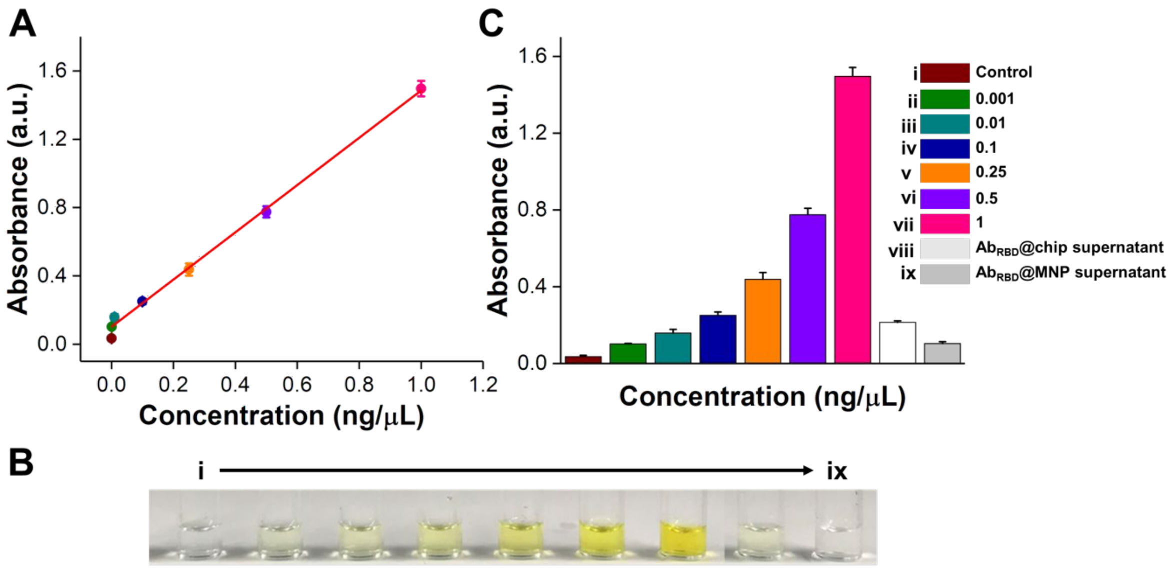

Supernatant	A_{450} (a.u.) Concentration (ng/μL)	Residual amount (ng)	Grafting amount (ng)	Grafting Rate (%)
Ab_{RBD}@chip	0.214<br>0.0813	81.3	168.64 ± 4.47	67.48 ± 1.79
Ab_{RBD}@MNP	0.103<br>0.0008	0.8	249.13 ± 5.81	99.68 ± 2.33

Figure S5. (A) The linear calibration curve between the A_{450nm} and the concentration of SH-Ab_{RBD} based on ELISA (n = 6). (B) Visual representation of SH-Ab_{RBD} standards (i-vii) and the supernatant samples from the Ab_{RBD}@chip (viii) and Ab_{RBD}@MNP (ix) for immobilization efficiency analysis. Alongside this visual, a table offers detailed quantifications. (C) A bar graph illustrating the absorbance levels from (B).

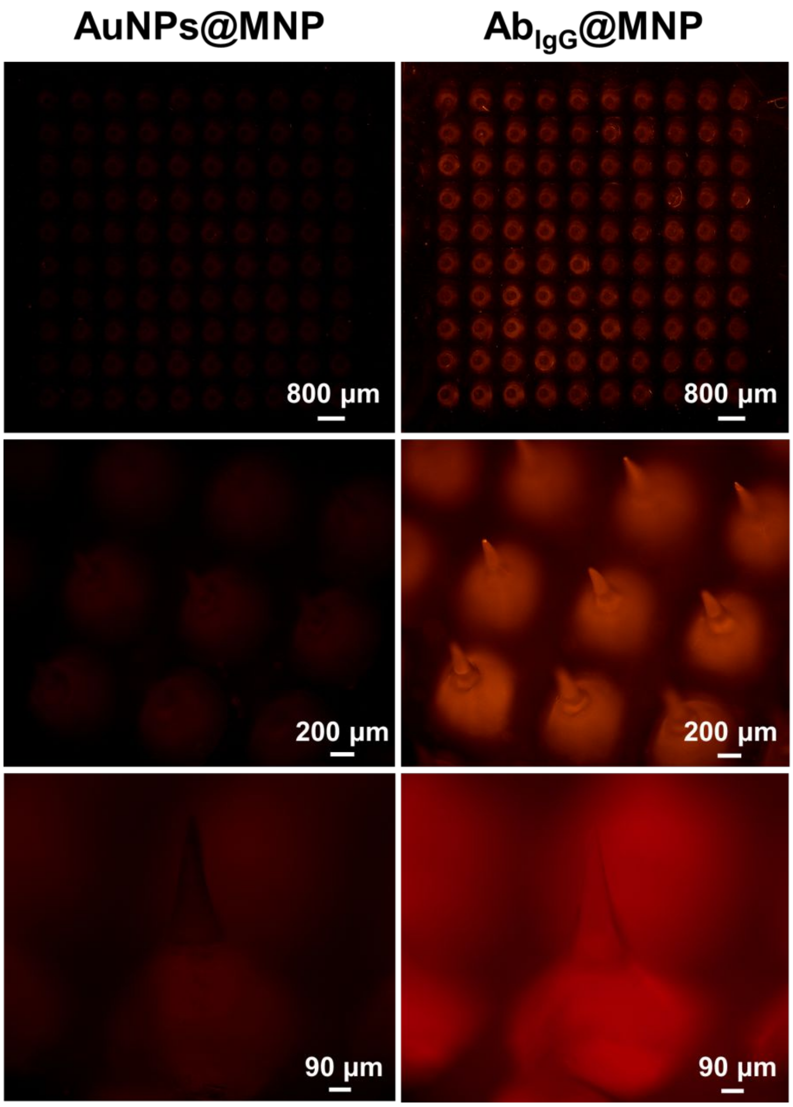

Figure S6. The distribution of biorecognition elements conjugated on MNP was assessed using a fluorescence microscope. ABflo® 647-labeled rabbit anti-rat IgG antibody (Ab_{IgG}) was conjugated to the MNP, forming Ab_{IgG}@MNP, which was then stained with ABflo® 647-labeled goat anti-rabbit IgG antibody.

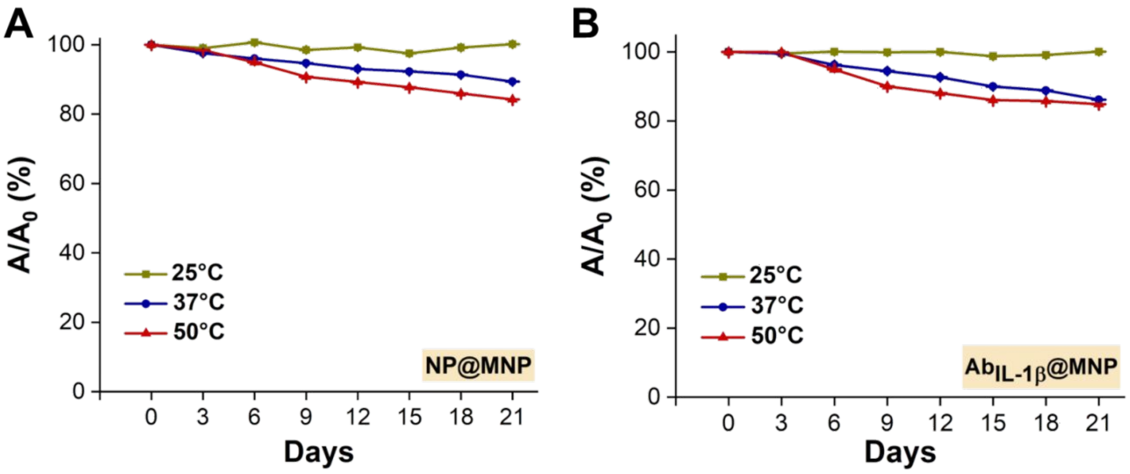

Figure S7. The long-term stability and durability of NP@MNP and Ab$_{IL-1β}$@MNP were assessed after storage under different temperature conditions (25, 37, and 50°C) for 3, 6, 9, 12, 15, 18, and 21 days. Detection efficiency was evaluated by measuring the A_{450nm} values (A) for detecting fixed concentrations of anti-SARS-CoV-2 NP IgA antibody and IL-1β, and these values were compared to the initial A_{450nm} values (A_0). The stability of NP@MNP and Ab$_{IL-1β}$@MNP was calculated using the formula: A_{450nm} (A) / A_{450nm} (A_0) × 100%.

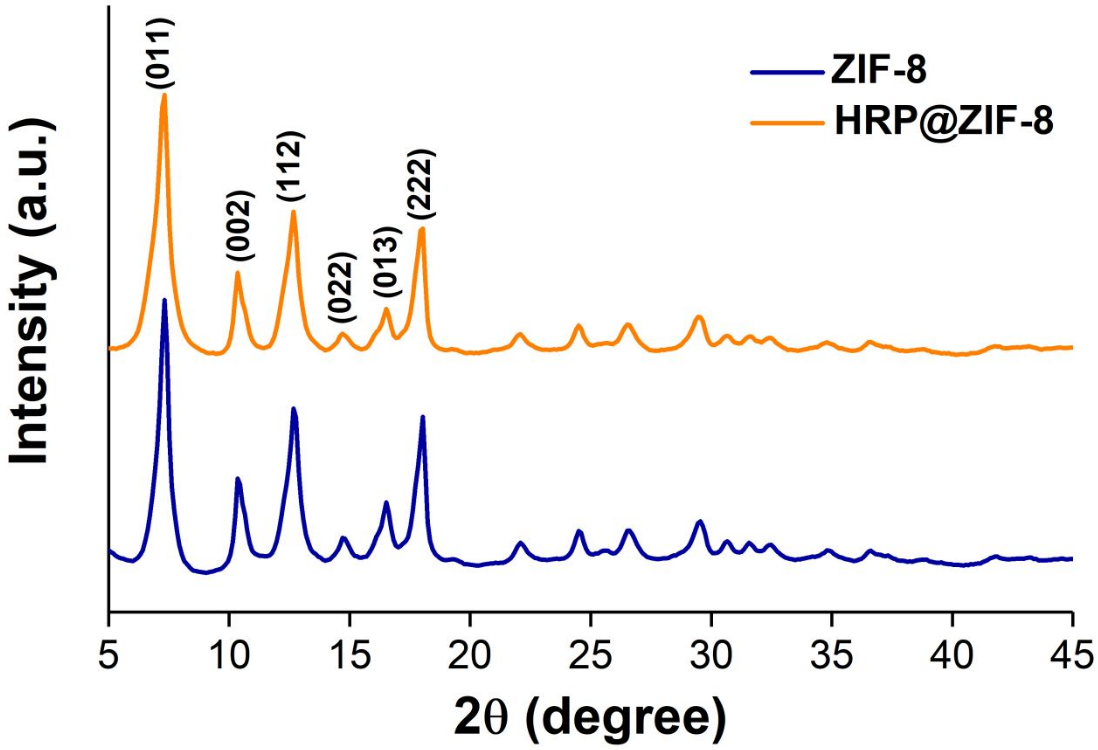

Figure S8. XRD pattern of ZIF-8 and HRP@ZIF-8. The XRD analysis on the prepared fillers is presented in the presence of solid peaks at 2θ = 7.3°, 10.4°, 12.7°, 14.7°, 16.5°, and 18.0° correspond to Miller indexes for planes of (011), (002), (112), (022), (013), and (222), respectively, which indicates high crystallinity of the prepared ZIF-8 and HRP@ZIF-8.

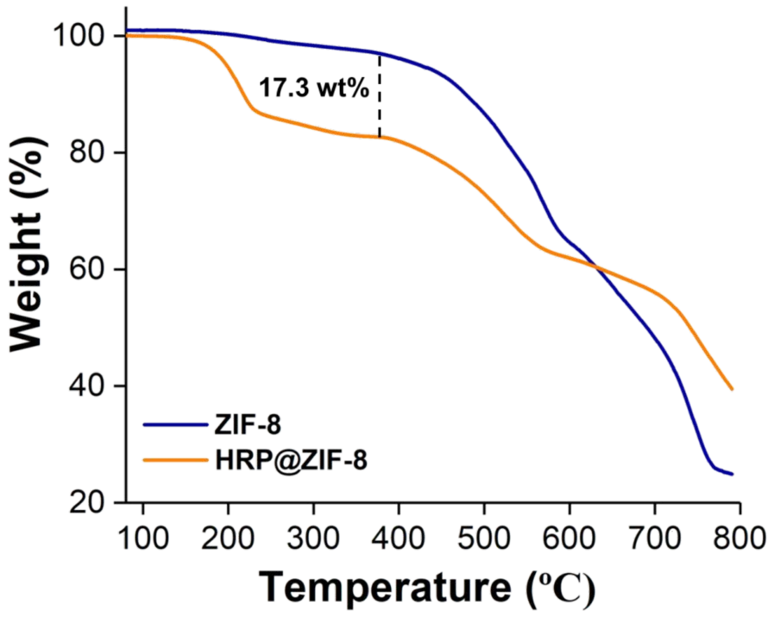

Figure S9. Thermogravimetric analysis (TGA) of the ZIF-8 samples synthesized via the bulk-solution methods in the absence and presence of HRP.

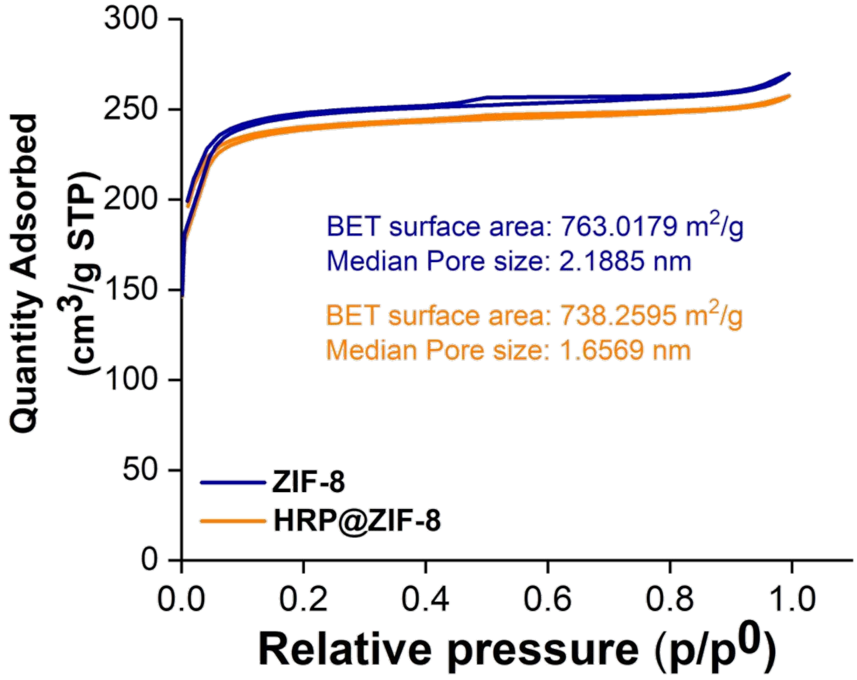

Figure S10. Nitrogen adsorption-desorption isotherms for ZIF-8 and HRP@ZIF-8 exhibit Type I behavior indicative of microporous structures, with a slight reduction in BET surface area and median pore size upon HRP encapsulation.

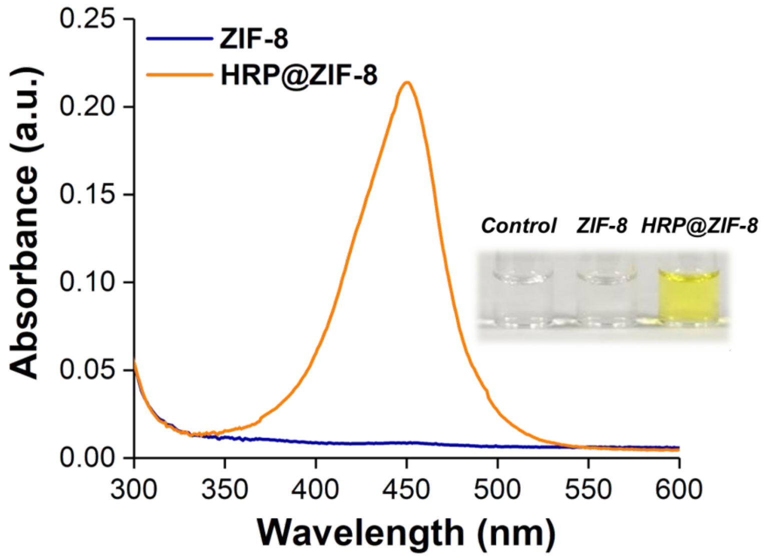

Figure S11. TMB assay of HRP encapsulated in ZIF-8 to confirm the successful formation of HRP@ZIF-8. The inset displays the image of ZIF-8 and HRP@ZIF-8 after a chromogenic reaction with TMB/H_2O_2.

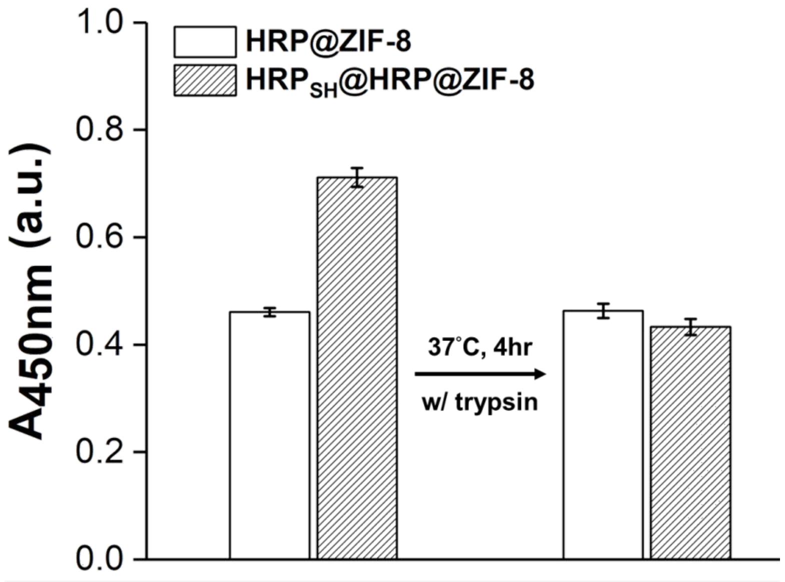

Figure S12. The catalytic activity of HRP@ZIF-8 and HRP_{SH}-HRP@ZIF-8 was analyzed before and after incubation with trypsin, using TMB/H_2O_2 as the substrate (n = 3).

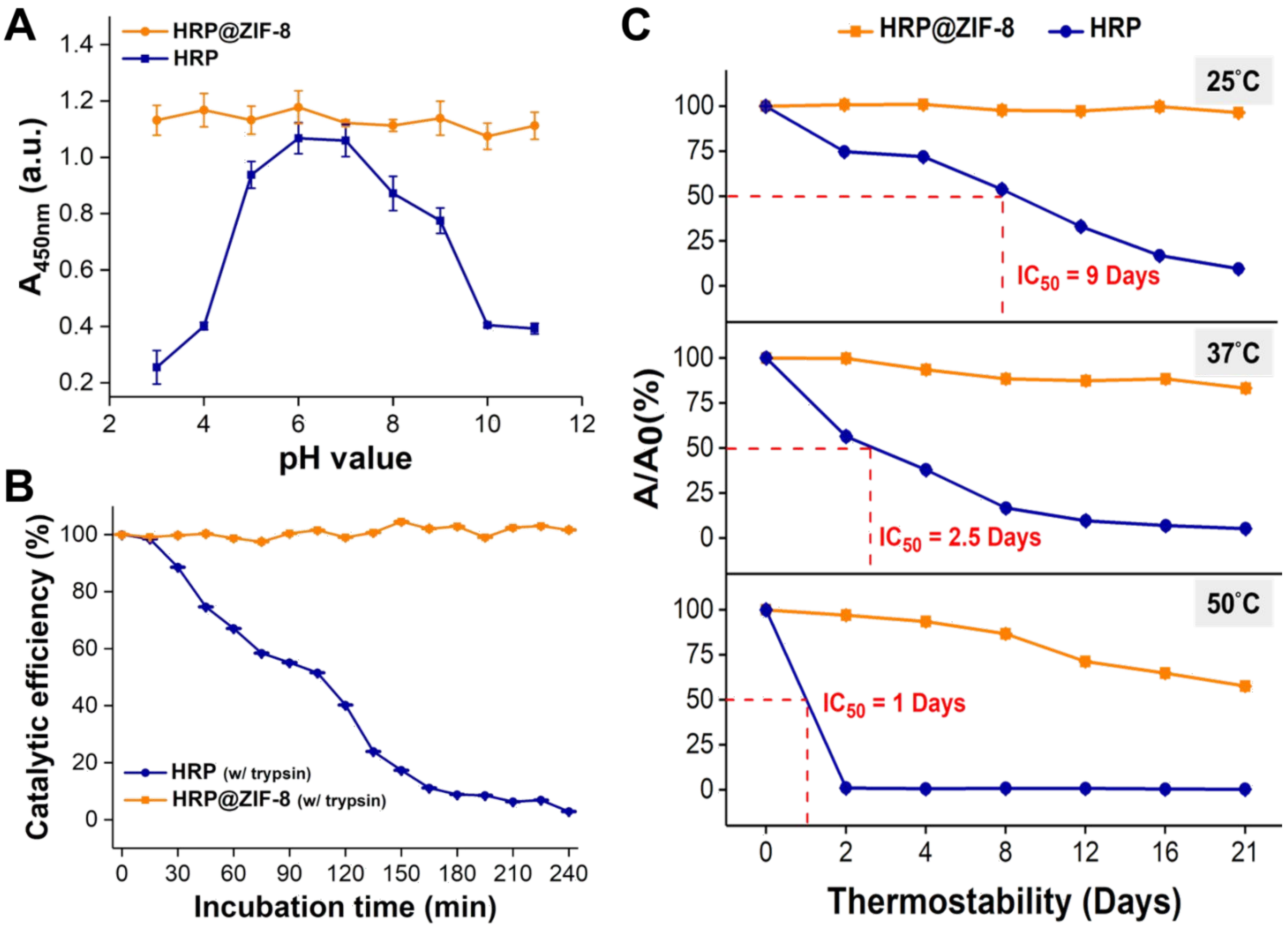

Figure S13. (A) The pH stability profile for naked HRP and HRP@ZIF-8 shows the remarkable pH resistance of HRP@ZIF-8, with stable catalytic activity across a broad pH range compared to naked HRP (n=3). (B) The stability profile for naked HRP and HRP@ZIF-8 demonstrates the remarkable trypsin resistance of HRP@ZIF-8, exhibiting stable catalytic activity over the incubation period compared to naked HRP (n = 3). (C) Comparison of thermostability between naked HRP and HRP@ZIF-8 at various temperatures (25°C, 37°C, and 50°C) reveals the superior thermal resistance of HRP@ZIF-8. It sustains significant enzymatic activity over 21 days when stored at 50°C (n=3).

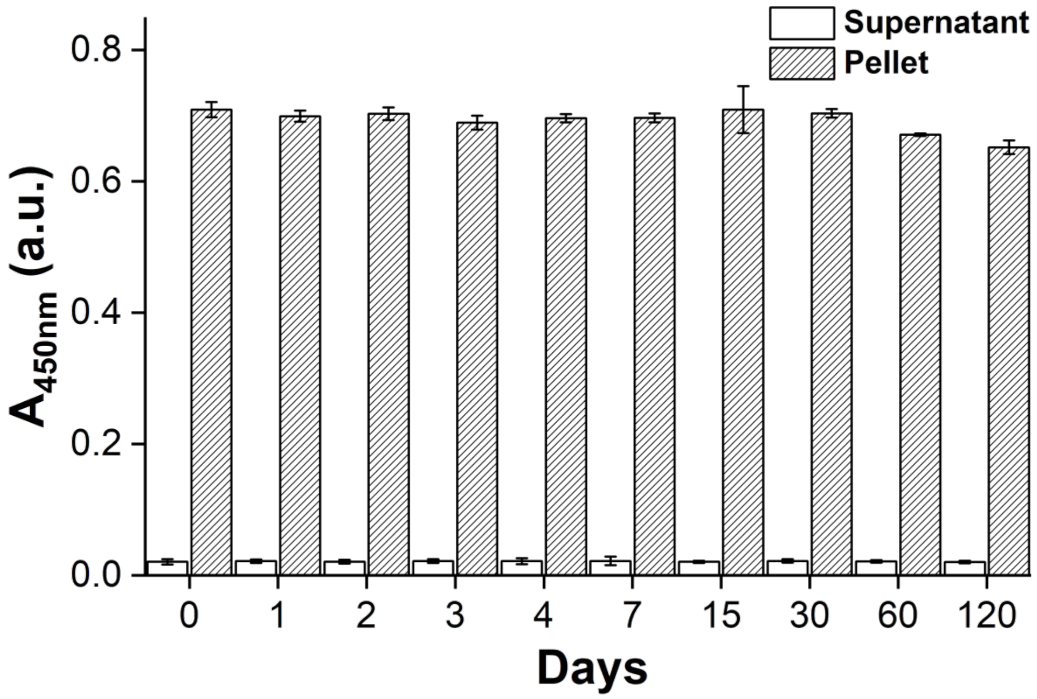

Figure S14. The stability of Ab_{RBD}-HRP@ZIF-8 in PBS was evaluated over time (t = 1, 2, 3, 4, 7, 15, 30, 60, and 120 days). At each time point, the samples were centrifuged, and the supernatants and pellets reacted with the TMB/H_2O_2 substrate. The A_{450nm} values obtained from the supernatants and pellets at each time point showed no significant difference compared to the initial A_{450nm} (t = 0). This indicates that the Ab_{RBD}-HRP@ZIF-8 remained intact and did not undergo decomposition in PBS.

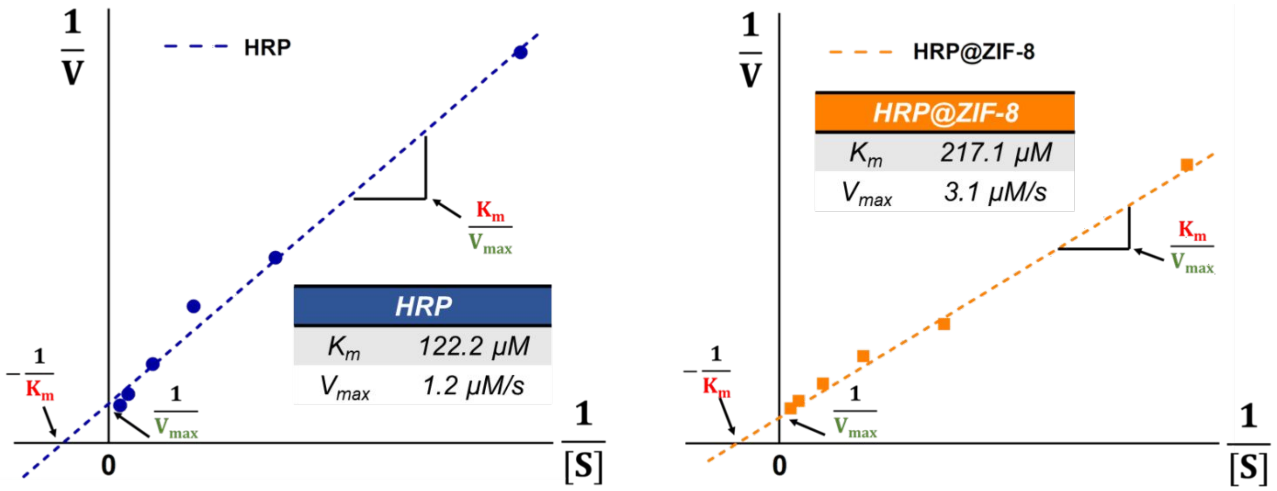

Figure S15. The Michaelis-Menten equation was used to determine the kinetic parameters of naked HRP and HRP@ZIF-8 via a Lineweaver–Burk plot. Here, V_0 represents the apparent initial reaction rate, [S] denotes the substrate (H_2O_2) concentration, V_{max} is the maximum rate achieved by the system at saturating substrate concentration, and K_m is the Michaelis–Menten constant.

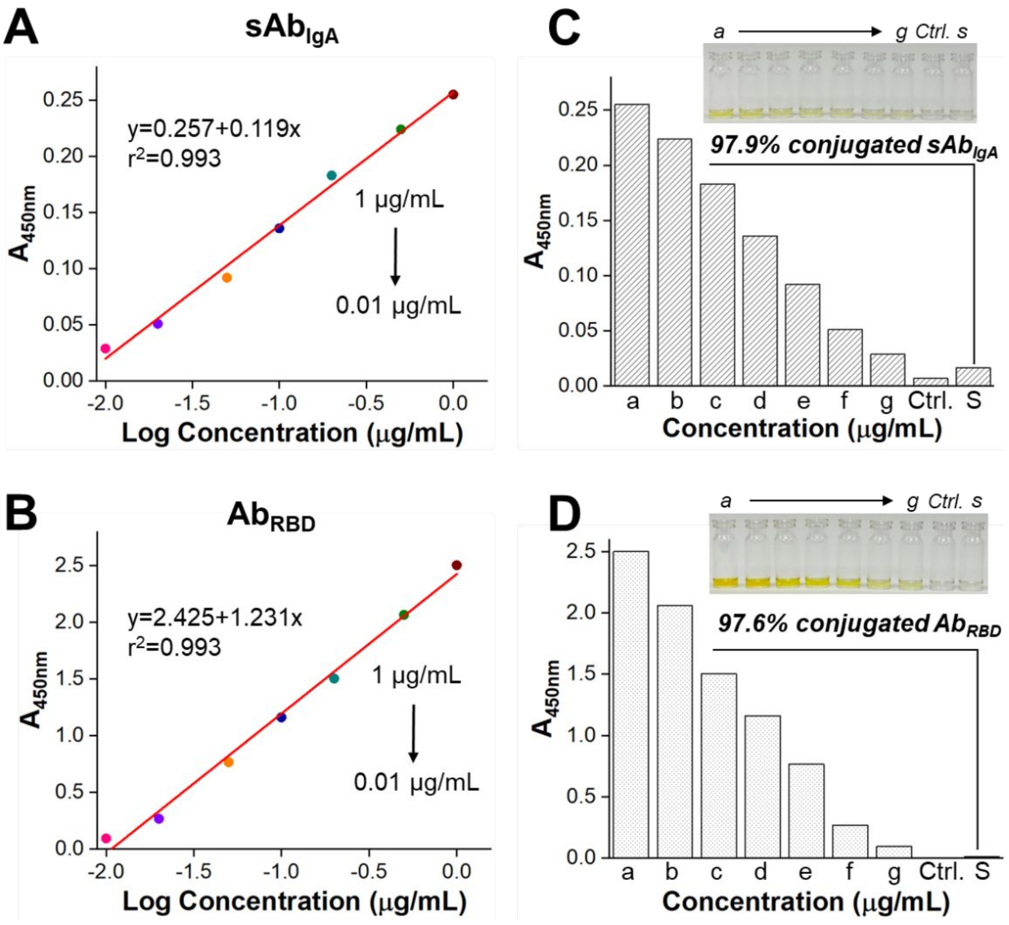

Figure S16. The evaluation of thiolated antibody conjugation onto HRP@ZIF-8 by ELISA assay. The linear regression of the standards for (A) sAb_{IgA} and (B) Ab_{RBD}, and the conjugation efficiency based on the analysis of the supernatants for (C) sAb_{IgA} and (D) Ab_{RBD}.

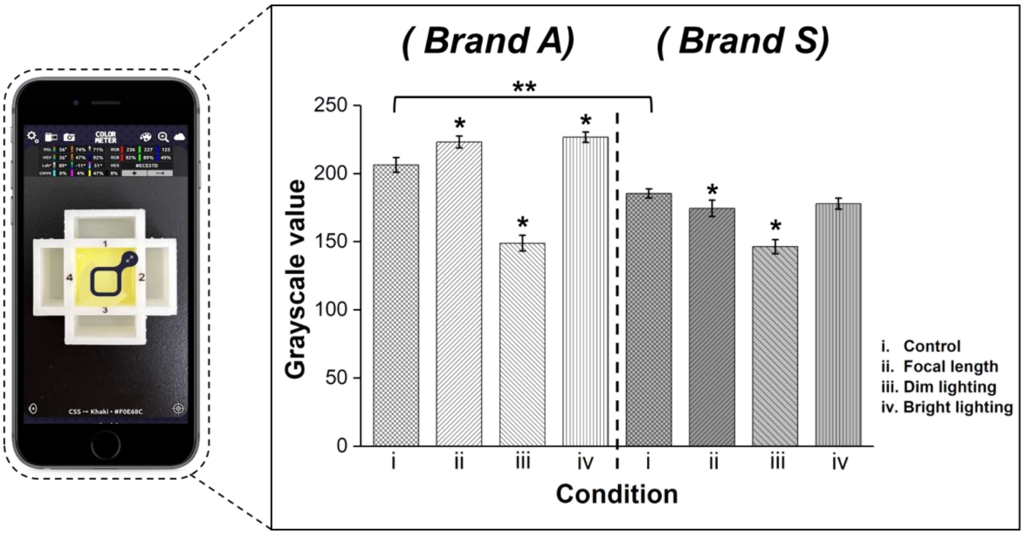

Figure S17. Grayscale value analysis of a fixed sample captured by brand A and S smartphones. (i) Control: Captured at a constant distance and under consistent lighting using a standard focal length. (ii) Focal length: Captured at a constant distance and under consistent lighting using double the standard focal length. (iii) Ambient light (Indoor): Captured at a constant distance and standard focal length under dim indoor lighting. (iv) Ambient light (Outdoor): Captured at a constant distance and standard focal length under bright outdoor lighting (n = 3). *indicates a significant difference compared to the control (Student's *t*-test, *$p \leq 0.05$); **indicates a significant difference (Student's *t*-test, **$p \leq 0.05$).

Table S1. The determination of spiked anti-SARS-CoV-2 NP IgA antibody, RBD, and SARS-CoV-2 Delta S1P in PVA solution (ranging from 1 wt% to 10 wt%, with viscosities from 100 to 1,000 cPs) was conducted using the MNP-based SenBox combined with a microplate spectrophotometer to record the A_{450nm} values and calculate the recovery rates (n = 9).

IgA (99.5%-105.1%) (n=9)

sample	Spiked conc. (pg/mL)	Found (pg/mL)	Recovery (%)	RSD (%)
1% PVA	50	49.74	99.48	7.14
	250	261.69	104.68	9.35
	1000	1051.36	105.14	9.04
5% PVA	50	50.55	101.09	6.51
	250	259.56	103.82	9.73
	1000	998.23	99.82	8.64
10% PVA	50	50.28	100.56	7.95
	250	255.51	102.21	6.51
	1000	1029.38	102.94	7.81

Spike-RBD (97.1%-102.4%) (n=9)

sample	Spiked conc. (pg/mL)	Found (pg/mL)	Recovery (%)	RSD (%)
1% PVA	50	50.82	101.63	9.77
	250	246.91	98.76	3.88
	1000	1023.45	102.35	4.01
5% PVA	50	48.55	97.11	9.53
	250	250.61	100.24	5.86
	1000	1017.28	101.73	4.54
10% PVA	50	51.02	102.05	8.97
	250	244.44	97.78	6.89
	1000	1005.76	100.58	4.24

Delta S1 (96.1%-105.6%) (n=9)

sample	Spiked conc. (pg/mL)	Found (pg/mL)	Recovery (%)	RSD (%)
1% PVA	50	49.88	99.75	8.53
	250	254.36	101.74	9.29
	1000	972.31	97.23	8.61
5% PVA	50	51.51	103.02	8.67
	250	257.42	102.97	6.15
	1000	1056.19	105.62	7.07
10% PVA	50	48.04	96.08	10.83
	250	259.25	103.71	9.61
	1000	978.32	97.83	6.56

Table S2. The specifications of Color Reader CR3.

Color Reader CR3	
**Measurement Mode:**<br>D/8 (Complies with national standards, 8° directional illumination, circular diameter for SCI)<br>**Light Spectrum:**<br>Full spectrum LED light source<br>**Light Source Life:**<br>5-years more than 3 million times measurements<br>**Measurement Aperture:** φ20mm<br>**Weight:** Approx. 88 g<br>**Wavelength:** 400 – 700 nm<br>**Measurement Interval:**<br>Response time in seconds<br>**Measurement Speed:** <1.0 s<br>**Observation Angle:** 2°/10°	**Color Spaces:**<br>CIE LAB, XYZ, Yxy, LCh, CIE LUV, s-RGB, HunterLab, βxy, DIN Lab99<br>**Color Difference Formula:**<br>ΔE*ab, ΔE*uv, ΔE*94, ΔE*cmc(2:1), ΔE*cmc(1:1), ΔE*00, DINΔE99, ΔE(Hunter)<br>**Stability:**<br>Within ΔE*ab 0.05 (Measure the whiteboard base, repeat every 5 min, average of 30 measurements)<br>**Other Color Index:**<br>WI(ASTM E313，CIE/ISO, AATCC, Hunter),<br>YI(ASTM D1925，ASTM 313), Metamerism Index Mt, Color fastness, color strength, and Opacity, 555 color classification, Munsell(C/2) (implemented by mobile APP)

Table S3. A comparison of anti-SARS-CoV-2 NP IgA antibody and SARS-CoV-2 S1P concentrations measured by the gold standard ELISA method and the MNP-based SenBox combined with a portable ColorReader.

Anti-SARS-CoV-2 NP IgA antibody		
U.S. Positive Saliva	Gold standard ELISA (pg/mL)	MNP-based SenBox (pg/mL)
Subject 1	211.11	217.42
Subject 2	43.33	58.21
Subject 3	104.44	98.38

SARS-CoV-2 S1P		
U.S. Positive Saliva	Gold standard ELISA (pg/mL)	MNP-based SenBox (pg/mL)
Subject 1	700.19	680.69
Subject 2	161.62	177.10
Subject 3	371.14	353.61

Table S4: Comparison of cytokine detection methods in this study with previously published works.

Study/Method	LOD (pg/mL)	Note
**This study (TNF-α / IL-1β)**	4.5 / 9.4	Fast, portable, and suitable for point-of-care use
**MILLIPLEX® Multiplex for Luminex® Immunoassays (Cytokines)**	2.7	High-throughput, multi-detection, not suitable for point-of-care
**Quench-Release-Based Fluorescent Immunosensor* (TNF-α)**	123	Requires fluorescent spectrometers, higher LOD
**Conventional ELISA on MNP** (IL-6)**	53	Bulky instruments, not suitable for point-of-care
**Fluor-linked immunosorbent assay on MNP** (IL-6)**	261	Requires fluorescent spectrometers, higher LOD
**Plasmonic fluor-linked immunosorbent assay on MNP** (IL-6)**	0.33	Highly sensitive, but requires spectrometers
**Electrochemical detection on MNP*** (IL-6)**	0.54	Real-time, highly sensitive, but requires electrochemical instruments

References:

* Li, H.; Li, X.; Chen, L.; Li, B.; Dong, H.; Liu, H.; Yang, X.; Ueda, H.; Dong, J. Quench-Release-Based Fluorescent Immunosensor for the Rapid Detection of Tumor Necrosis Factor α. *ACS Omega* **2021**, *6* (46), 31009-31016.

** Wang, Z.; Luan, J.; Seth, A.; Liu, L.; You, M.; Gupta, P.; Rathi, P.; Wang, Y.; Cao, S.; Jiang, Q.; et al. Microneedle patch for the ultrasensitive quantification of protein biomarkers in interstitial fluid. *Nat. Biomed. Eng.* **2021**, *5* (1), 64-76.

*** Xu, J.; Yang, B.; Kong, J.; Zhang, Y.; Fang, X. Real-Time Monitoring and Early Warning of a Cytokine Storm In Vivo Using a Wearable Noninvasive Skin Microneedle Patch. *Adv. Healthc. Mater.* **2023**, *12* (18), 2203133.

Table S5. Comparison of TNF-α and IL-1β concentrations quantified by a commercial spectrophotometer and a portable ColorReader.

In vivo

TNF-α			IL1-β		
Samples	***Spectrometer (pg/mL)***	***ColorReader (pg/mL)***	***Samples***	***Spectrometer (pg/mL)***	***ColorReader (pg/mL)***
N/C	0.00	0.00	N/C	0.00	0.00
1a	0.00	2.10	1a	0.00	3.81
2a	0.00	2.80	2a	0.00	2.75
3a	621.77	655.33	3a	1618.90	1717.31
4a	313.99	330.29	4a	1388.90	1450.05
5a	309.54	368.35	5a	1618.90	1647.90
6a	1241.77	1348.42	6a	2413.90	2682.66
1b	0.00	2.64	1b	0.00	2.23
2b	0.00	0.87	2b	0.00	2.23
3b	0.00	2.81	3b	0.00	7.17
4b	0.00	6.82	4b	0.00	9.25
5b	0.00	3.73	5b	0.00	6.58
6b	0.00	4.82	6b	0.00	9.93
